# Differing associations between sex determination and sex‐linked inversions in two ecotypes of *Littorina saxatilis*

**DOI:** 10.1002/evl3.295

**Published:** 2022-08-12

**Authors:** Katherine E. Hearn, Eva L. Koch, Sean Stankowski, Roger K. Butlin, Rui Faria, Kerstin Johannesson, Anja M. Westram

**Affiliations:** ^1^ Ecology and Evolutionary Biology, School of Biosciences University of Sheffield Sheffield S10 2TN United Kingdom; ^2^ Department of Zoology University of Cambridge Cambridge CB2 3EJ United Kingdom; ^3^ ISTA (Institute of Science and Technology Austria) Klosterneuburg 3400 Austria; ^4^ Department of Marine Sciences University of Gothenburg Strömstad SE‐45296 Sweden; ^5^ CIBIO, Centro de Investigação em Biodiversidade e Recursos Genéticos, InBIO Laboratório Associado Campus de Vairão, Universidade do Porto Vairão 4485‐661 Portugal; ^6^ BIOPOLIS Program in Genomics, Biodiversity and Land Planning, CIBIO Campus de Vairão, Universidade do Porto Vairão 4485‐661 Portugal; ^7^ Faculty of Biosciences and Aquaculture Nord University Bodø 8026 Norway

**Keywords:** Hybrid zone, local adaptation, recombination suppression, sex chromosomes, sexual antagonism

## Abstract

Sexual antagonism is a common hypothesis for driving the evolution of sex chromosomes, whereby recombination suppression is favored between sexually antagonistic loci and the sex‐determining locus to maintain beneficial combinations of alleles. This results in the formation of a sex‐determining region. Chromosomal inversions may contribute to recombination suppression but their precise role in sex chromosome evolution remains unclear. Because local adaptation is frequently facilitated through the suppression of recombination between adaptive loci by chromosomal inversions, there is potential for inversions that cover sex‐determining regions to be involved in local adaptation as well, particularly if habitat variation creates environment‐dependent sexual antagonism. With these processes in mind, we investigated sex determination in a well‐studied example of local adaptation within a species: the intertidal snail, *Littorina saxatilis*. Using SNP data from a Swedish hybrid zone, we find novel evidence for a female‐heterogametic sex determination system that is restricted to one ecotype. Our results suggest that four putative chromosomal inversions, two previously described and two newly discovered, span the putative sex chromosome pair. We determine their differing associations with sex, which suggest distinct strata of differing ages. The same inversions are found in the second ecotype but do not show any sex association. The striking disparity in inversion‐sex associations between ecotypes that are connected by gene flow across a habitat transition that is just a few meters wide indicates a difference in selective regime that has produced a distinct barrier to the spread of the newly discovered sex‐determining region between ecotypes. Such sex chromosome‐environment interactions have not previously been uncovered in *L. saxatilis* and are known in few other organisms. A combination of both sex‐specific selection and divergent natural selection is required to explain these highly unusual patterns.

**Impact Summary**

Sexual antagonism is believed to be one of the leading drivers of the evolution of sex chromosomes. Recombination suppression helps to resolve conflict in the genome between the sexes. Chromosomal inversions may be involved but their specific role remains unclear. Inversions are also known to contribute to local adaptation and this selection may interact with sexually antagonistic selection in complex ways. Population comparisons in species with young sex chromosomes are needed to disentangle these processes. We use genetic data to study a Swedish population of a marine snail, *Littorina saxatilis*, which is split into two distinct but connected ecotypes on different parts of the rocky shore. In one ecotype, we find new evidence for a sex‐determining region where females are heterozygous (ZW system). This sex chromosome is covered by four putative inversions that show differing associations with sex. Dramatic differences occur between the ecotypes. The same inversions are present at different frequencies in the other ecotype but are not associated with sex; no sex‐determining region is detectable. We discuss potential combinations of both sexually antagonistic selection and divergent natural selection between the ecotypes that could have produced the observed patterns. Our results demonstrate the value of intraspecific comparisons and provide insight into how sex chromosome evolution and adaptive divergence may interact.

Species with separate sexes experience evolutionary challenges because males and females are subject to different patterns of selection (Connallon 2015) and, therefore, fitness effects of some alleles differ between the sexes (Connallon & Clark 2014). The appearance of such sexually antagonistic alleles, followed by suppression of recombination to link sexually antagonistic loci and the sex‐determining locus to avoid fitness cost, is a common hypothesis for driving the evolution of sex chromosomes (Fisher 1931; Rice 1987; Rice 1996; Wright et al. 2016), although alternative models are available (e.g., Lenormand and Roze 2022). Despite an increase in research into nascent sex chromosomes and interspecies comparisons, it remains challenging to test models for the drivers of sex chromosome evolution (Wright et al. 2016; Abbott et al. 2017; Vicoso 2019; Furman et al. 2020). For example, sex chromosomes are the most advantageous location in the genome for the emergence of sexually antagonistic alleles, so it is unclear whether these loci drive sex chromosome evolution or accumulate after chromosome differentiation (Rice 1984; Charlesworth et al. 2005). Chromosomal inversions are one possible mechanism for impeding recombination in the heterogametic sex (Charlesworth 1991). Inversions on sex chromosomes have been observed in a number of taxa, including birds (Wang et al. 2014; Wright et al. 2014), primates (Lahn and Page 1999; Shearn et al. 2020), fish (Natri et al. 2019), snakes (Vicoso et al. 2013), and papaya (Wang et al. 2012). However, inversions can be a consequence of, rather than a mechanism for, recombination suppression (e.g., *Neurospora tetrasperma*; Sun et al. 2017). Lack of recombination due to other means removes selection for gene order, allowing structural variants such as inversions to accumulate (Furman et al. 2020). Species with young, emerging sex chromosomes are likely to be valuable systems for addressing such questions, because it is possible to make intraspecific comparisons where the genomic basis of sex is labile. Few studies have used this opportunity (Furman et al. 2020).

Sex chromosome evolution is usually assumed to occur in a homogenous environment. In reality, environments, populations, and patterns of selection are heterogeneous in space and time. The potentially complex effects of this heterogeneity on sex chromosome evolution are important, but greatly understudied (Abbott et al. 2017). For example, divergent selection may drive frequency differences of inversion arrangements that suppress recombination between loci for locally adaptive traits and are therefore useful for local adaptation (Kirkpatrick & Barton 2006; Joron et al. 2011; Wang et al. 2013; Lee et al. 2017). However, it remains unknown if the same inversions are associated with sex chromosome evolution and local adaptation, and whether the two different processes interact.

There are clear similarities between the processes of adaptive divergence and sex chromosome evolution. In both cases, inversions are thought to maintain beneficial combinations of alleles at different loci (Charlesworth 2016; Huang & Rieseberg 2020). The two processes might interact if a driver of sex chromosome evolution, for example, sexual antagonism, was environment dependent and so drives differential evolution of sex chromosomes between populations (Bracewell et al. 2017; Wright et al. 2017; Lasne et al. 2018). Traits such as size (e.g., in *Littorina saxatilis;* Perini et al. 2020) or color (e.g., in guppies; Wright et al. 2017) that are important in one sex for mate choice may also confer greater negative fitness effects in certain environments according to strength of selection pressures such as predation. For example, a young sex chromosome could influence gene flow with a connected population that does not experience sexual antagonism, or an inversion that captures locally adapted alleles may also capture the sex‐determining locus and so inhibit the spread of a nascent sex chromosome into other environments.

With these processes in mind, we investigated sex determination in a well‐studied example of local adaptation with gene flow, the intertidal snail *L. saxatilis* (Johannesson et al. 2020). The species is ovoviviparous, contributing to low lifetime dispersal, which has facilitated local adaptation over small spatial scales (Reid 1996). Two distinct ecotypes have adapted to differing rocky shore habitats (Johannesson et al. 2010; Butlin et al. 2014). In Sweden, the Crab ecotype inhabits boulder fields and has evolved to withstand crab predation. It has a larger, thicker, elongated shell with a relatively smaller aperture and is more wary in its behavior (Fig. 1) (Johannesson et al. 2010). The Wave ecotype is adapted to withstand wave action on rocky headlands via a smaller, thinner, globose shell that allows sheltering in small crevices (Fig. 1) (Johannesson et al. 2010). Despite this ecological selection and some degree of habitat and mate choice (Johannesson et al. 2010), the ecotypes readily hybridize (Panova et al. 2006; Hollander et al. 2015; Westram et al. 2018).

**Figure 1:**
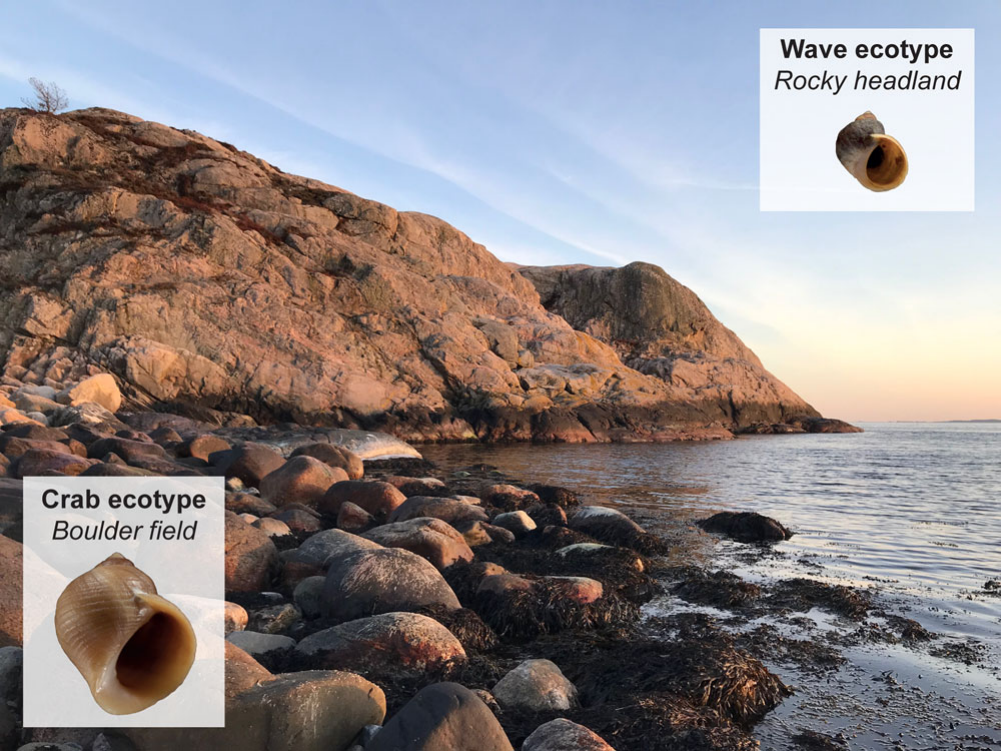
Image of the Crab‐Wave ecotype transition sampled on Ängklåvebukten. The hybrid zone is the area where the boulder field and rocky cliff habitats meet. Inset are images of typical Crab and Wave individuals of *Littorina saxatilis*.

Genetic and phenotypic clines between the ecotypes are replicated at many locations across the species range (Grahame et al. 2006; Galindo et al. 2019; Westram et al. 2021). Multiple putative inversions have been identified in *L. saxatilis*, some of which show systematic frequency differences between the ecotypes and are associated with adaptive traits (Westram et al. 2018; Faria et al. 2019; Morales et al. 2019; Koch et al. 2021; Westram et al. 2021). *Littorina saxatilis* has separate sexes that are genetically determined (Fretter & Graham 1962). However, strongly heteromorphic sex chromosomes have not been observed, leaving the sex‐determination mechanism unknown (García‐Souto et al. 2018). Sexual dimorphism has been identified in reproductive anatomy (Fretter & Graham 1962) and traits such as size and shape (Larsson et al. 2020). Size‐assortative mating creates sexual selection for a smaller male size (Perini et al. 2020). A recent study using crosses of Crab and Wave ecotypes of Swedish *L. saxatilis* found a strong quantitative trait locus (QTL) for sex on one linkage group (LG12) (Koch et al. 2021) but did not characterize the sex‐determination system. Combined with the knowledge of multiple putative inversions (Faria et al. 2019), including two on LG12 (that showed frequency differences between ecotypes but were not tested for associations with sex), this makes *L. saxatilis* an ideal system to study the interaction between sex chromosome evolution and local adaptation within a species.

Here, we test for the presence of a sex‐determining region in *L. saxatilis* through analysis of sex‐specific patterns in SNP data from a transect of snails across a hybrid zone in Sweden. We find evidence for a female heterogametic sex chromosome system, but only in the part of the transect that is inhabited by the Crab ecotype. Almost the entire length of LG12 is spanned by four putative inversions, but they show varying levels of sex and ecotype differentiation. They may represent distinct strata of a nonrecombining region whose evolution has apparently been influenced by barriers to gene flow between ecotypes.

## Methods

### SAMPLING AND GENOTYPING

This study used a dataset previously published in Westram et al. (2018). Sampling and data generation methodology are described in brief here; for full details, see Westram et al. (2018).

Six hundred snails were sampled along a transect that crossed the Crab‐Wave ecotype transition from boulder field to rocky cliff at Ängklåvebukten (Swedish west coast; 58°52ʹ15.14″N 11°07ʹ11.88″E; Fig. 1). Snail positions were recorded in three dimensions. Positions were subsequently collapsed to a one‐dimensional path to facilitate cline analysis. Size, shape, and sex were determined for each snail. DNA was extracted from 373 sexually mature snails as described in Panova et al. (2016), before capture sequencing using 40,000 120‐bp probes, randomly distributed across the genome. Read mapping to the reference genome (Westram et al. 2018), quality control, filtering, and genotyping were conducted as described in Westram et al. (2018). The only difference in this study was during the generation of an additional VCF for LG12, with the exclusion of the –variants‐only argument in the command bcftools (version 1.11) call, and reducing minimum alleles required from 2 to 1 during VCF filtering. This resulted in an all‐sites VCF including invariant sites and SNPs, covering 12,355 kbp (on LG12) of the 1.35 Gbp genome (all 17 LGs).

### DATA PREPARATION

All analyses were performed using R (version 4.0.0; R Core Team 2021) and the packages dplyr (version 1.0.5; Wickham et al. 2021) and ggplot2 (version 3.3.0; Wickham 2016) unless otherwise stated. Only genotyped snails were used (205 females and 168 males). For some analyses, the position of the snail on the transect, relative to the main environmental transition at 78 m (Westram et al. 2018), was used to classify snails by ecotype: <68 m, Crab; 68–88 m, hybrid; >88 m, Wave. For analyses that required exclusion of hybrids, 64 male and 57 female hybrids were removed to leave a total of 252 snails: 90 Crab females, 62 Crab males, 58 Wave females, and 42 Wave males. Greater numbers of females were likely due to a sampling bias toward larger individuals (Perini et al. 2020), rather than a sex‐ratio bias in the population.

### DETECTION OF A SEX‐ASSOCIATED REGION

A QTL for sex (Koch et al. 2021) is located on linkage group 12 (LG12), and initial analyses for sex‐associated SNPs (see below) yielded only SNPs on LG12. Therefore, of the set of SNPs produced from the capture sequencing, only SNPs located on contigs in the reference genome that mapped to LG12 were retained (linkage map from Westram et al. 2018). Eight contigs contained SNPs with more than one assigned map position; for this small number of SNPs, the most common map position for the contig was used. This gave a total dataset of 8657 SNPs with map positions located on 713 contigs on LG12.

Genotype and allele frequencies were calculated for each SNP, separately for each sex and ecotype. The frequency of heterozygotes for each SNP was compared between the sexes. SNPs in sex‐determining regions (linked to the sex‐determining locus, potentially with recombination suppression creating divergence between nascent Z and W chromosomes) are expected to diverge in genotype and allele frequencies between the sexes (Pucholt et al. 2017; Palmer et al. 2019). SNPs outside these regions are not expected to show significant differences between sexes. Deviations from this expectation were quantified by measuring residuals from the 1:1 relationship between proportions of heterozygotes in males and females (male heterozygosity minus female heterozygosity). These residuals were then plotted on the linkage map to indicate the position of the sex‐determining region.

Sex‐specific recombination maps were examined to detect any difference between the sexes. A sex‐determining region is expected to show recombination suppression in the heterogametic sex; recombination is also suppressed in individuals of either sex that are heterozygous for an inversion. Maps were available from a Crab × Crab cross (Westram et al. 2018) and from Crab × Wave crosses (Koch et al. 2021), both using individuals from the population sampled in our transect. The Crab × Wave maps were produced from several families whose parents were Crab × Wave hybrids, whereas the Crab × Crab map was a product of a single pair of parents. Unless otherwise stated, the sex‐averaged Crab × Crab map was used to position contigs.

### AN ECOTYPE LIMITED, SEX‐ASSOCIATED REGION

When heterozygote proportions were compared between the sexes, only LG12 showed strong sex differences and as such only LG12 was retained for further analysis. However, separate examination of heterozygosity on LG12 in each ecotype revealed that sex differences were limited to the Crab ecotype (see below). As a result, comparisons of heterozygosity were repeated for all other linkage groups with only Wave individuals to test for a sex‐associated linkage group in this ecotype that may have been masked when both ecotypes were analyzed together. A total of 255,114 SNPs with map positions on 11,155 contigs across the 17 linkage groups were used. The distribution across the 17 linkage groups of the 1% of SNPs with the most negative residuals was used to test for a sex‐associated linkage group.

### INVERSION DETECTION USING LINKAGE DISEQUILIBRIUM AND PRINCIPAL COMPONENT ANALYSES

A similar methodology to the one used in Faria et al. (2019) to detect putative chromosomal inversions in *L. saxatilis* was implemented here for LG12. These analyses exploit the expectation of high linkage disequilibrium (LD) for loci in polymorphic inversions, compared to surrounding regions. Because inversions across the sex‐determining region may differ between males and females, LD analysis of both sexes together may mask detection of groups of SNPs that are in high LD in one sex only. Therefore, only females were used for the LD analysis (male data were included in the next step of cluster investigation). All females from across the transect were included.

Briefly, the package genetics (version 1.3.8.1.2; Warnes et al. 2019) was used to generate a matrix of pairwise LD (*r* ^2^) values for all SNPs. This LD matrix was then used with the package ldna (version 0.6.4; Kemppainen et al. 2015) to identify “outlier clusters” of SNPs that showed higher LD than the rest of the linkage group. The package allows variation in two parameters that affect the detection of clusters: |*E*|_min_ and φ. These were manipulated, similarly to in Faria et al. (2019), to produce a set of outlier clusters of interest (see *Methods* in the Supporting Information for details of parameter combinations and criteria).

To investigate the clusters of SNPs in high LD identified by LDna, principal component analysis (PCA) was used. When high LD clusters are generated by inversions, SNPs are expected to be clustered in one region of a linkage group and a PCA of that region groups individuals by inversion genotype (two homozygote groups and one heterozygote group, if two arrangements are present). Other causes of high LD clusters are unlikely to share these properties (see Kemppainen et al. [2015] and Faria et al. [2019] for a more detailed discussion). Therefore, we examined the position of SNPs in each cluster on the LG12 genetic map and performed PCA on all SNPs (not just those in the LD cluster) in each cluster region. PCA was carried out using R packages hmisc (version 4.4.0; Harrell Jr 2020) and adegenet (version 2.1.3; Jombart 2008; Jombart & Ahmed 2011) on the male and female data together.

### CLINE FITTING

Cline analysis was conducted for putative inversions identified on LG12 to examine changes in frequency across the hybrid zone from Crab to Wave and any differences between the sexes. Clines were fitted for the putative inversion arrangement that was more frequent in Crab than Wave in females (or in males when the frequency in females did not vary). This arrangement was labeled R (reference arrangement), and the other A (alternate).

Clines were fitted to putative inversion genotypes across the transect using a simple sigmoid model, following the formulation from Derryberry et al. (2014), and using the mle2 function in the R package bbmle (version 1.0.23.1; Bolker and R Development Core Team 2020). Five models were fitted: a null model (no change in arrangement frequency), the full model (separate parameters for male and female center, width, Crab and Wave frequencies), and three constrained models to test parameter differences between males and females: “combined” (all parameters equal between sexes), “constrained” (only center and width equal between sexes), and “Wave‐constrained” (center, width and Wave frequency equal between sexes). No Crab‐constrained model was included as sex differences in arrangement frequency are expected in Crab for sex‐linked inversions. We also considered the possibility of no cline in one sex and a cline in the other. The AIC of each model was used to test which best fitted the data and therefore whether cline parameters differed between the sexes. For illustration, arrangement frequencies for each putative inversion were calculated along the transect, for each sex, in overlapping sliding windows of 25 snails shifting by five snails.

### DIVERGENCE AND DIVERSITY ESTIMATES

Genetic diversity (π) and divergence (*d*_XY_) were calculated for putative inversion genotypes for an insight into the age and sequence of evolution of the inversions. An all‐sites VCF was used for calculation of these statistics because this is known to reduce bias in the estimates. Calculations of π and *d*_XY_ were carried out separately for each putative inversion using custom scripts from Martin (2020). Individuals were split into three groups according to putative inversion genotype (heterozygotes and the two homozygote groups) and also by sex and ecotype (giving up to 12 “populations” when the three genotypes were present in both sexes and ecotypes). π was calculated within each of these groups and *d*_XY_ between each pair of groups. Because the reference genome for *L. saxatilis* is not contiguous, statistics were calculated for each contig by setting a nonoverlapping window size of 2000 bp; a small number of large contigs were split into two or three windows using this window size.

*d*_XY_ between inversion arrangements was calculated using the π values for inversion genotypes using the following equation:

$$d_{\mathrm{XY}}\;\mathrm{between}\;A\;\mathrm{and}\;R\;=\;2\left(\pi_{\mathrm{RA}}-\frac{\pi_{\mathrm{RR}}}{4}-\frac{\pi_{\mathrm{AA}}}{4}\right),$$

where π_RA_, π_RR_, and π_AA_ are the nucleotide diversities for the heterokaryotypes and two groups of homokaryotypes, respectively. This equation makes allowance for the fact that half of the comparisons in the heterozygotes are between arrangements and the other half are within one arrangement or the other.

To test the effect of genotype, sex, and ecotype on nucleotide diversity in putative inversion arrangements, mixed models were fitted to the π values calculated for groups of individuals of each combination of these variables, separately for each putative inversion, using lme4 (version 1.1.27; Bates et al. 2015) and MuMIn (version 1.43.17; Barton 2009). See *Methods* in the Supporting Information for details.

### INVERSION GENOTYPE‐GENOTYPE AND GENOTYPE‐SEX ASSOCIATIONS

Associations between genotypes at different putative inversions, and between each putative inversion and sex, were assessed using chi‐square contingency tests in the packages zoo (version 1.8.8; Zeileis & Grothendieck 2005) and tidyquant (version 1.0.3; Dancho 2021). This was carried out separately for each ecotype. Squared correlation coefficients were used to measure the strength of association.

## Results and Discussion

Our data suggest a female‐heterogametic (ZW) sex determination system in the Crab ecotype of *L. saxatilis* at our study site in Sweden. The sex chromosome pair contains four regions of suppressed recombination, consistent with putative chromosomal inversions, some of which behave like strata on the sex‐specific (W) chromosome. However, these putative inversions are not associated with sex in the Wave ecotype at the same site and the sex determination system for the Wave ecotype remains uncertain. Below, we present the evidence that leads to these novel conclusions and then consider scenarios that might have led to the different patterns between the ecotypes.

### FEMALE‐HETEROGAMETIC SEX DETERMINATION IN THE CRAB ECOTYPE

**Figure 2:**
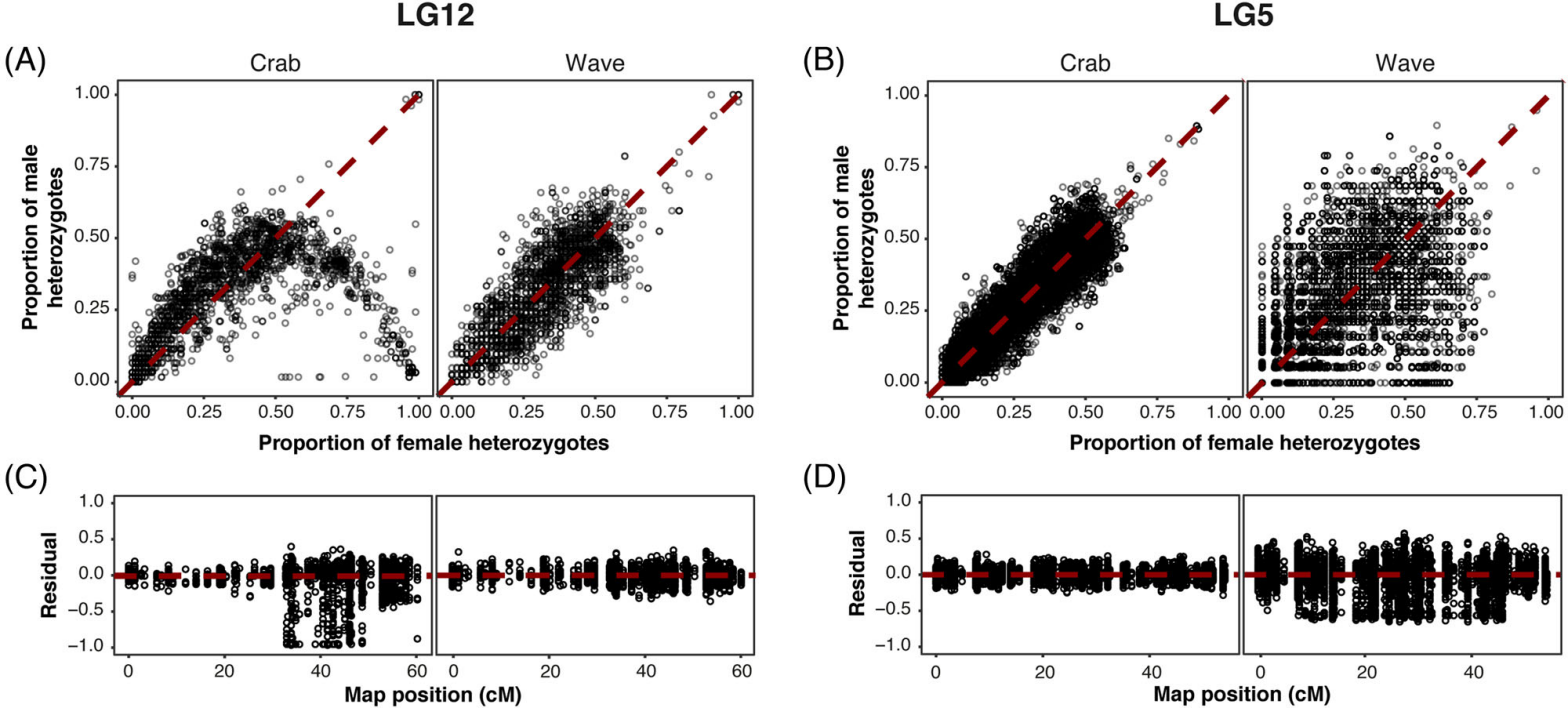
The proportions of each sex that are heterozygous at SNPs on (A) LG12 and (B) LG5 in the two ecotypes. SNPs with a greater difference in heterozygosity between the sexes are further from the 1:1 line (neutral expectation of equal heterozygote proportions between the sexes). The distribution of sex‐associated SNPs on (C) LG12 and (D) LG5 along their respective genetic maps. Residuals quantify the deviation of SNPs from neutral expectation, calculated as female heterozygosity – male heterozygosity.

Association of genotypes with sex can be one of the first indicators of the evolution of a young sex‐determining region (Pucholt et al. 2017; Palmer et al. 2019). In our data, although most LG12 SNPs followed the neutral expectation of equal proportions of heterozygotes in each sex, a group of SNPs departed strongly from this expectation (Fig. 2A). In these deviating SNPs, heterozygosity was skewed toward females but few were heterozygous in all females suggesting that they are linked to, rather than at, a sex‐determining locus. SNPs showed varying strengths of association with sex as reflected by the continuous distributions of heterozygosity (Fig. 2A) and residuals (Fig. 2C). There was a striking difference between the two ecotypes: Crab snails showed many sex‐associated SNPs, with some close to perfect association (all females heterozygous, all males homozygous) but there was no such association in the Wave individuals. These results indicate a ZW sex‐determining system in the Crab ecotype but provide no evidence concerning the Wave sex determination system.

Sex‐associated loci are expected when recombination has ceased in a region of the chromosome surrounding the sex‐determining locus, so that loci in this region build up LD with the sex‐determining locus (Abbott et al. 2017). Therefore, we checked how the sex‐associated SNPs were distributed along the genetic map for LG12, with the expectation that sex‐associated SNPs are clustered. The pattern described is for the Crab ecotype as sex‐associated SNPs were found only in this group. The first half of the linkage group up to 32.8 cM did not hold any sex‐associated SNPs (Fig. 2C). Nearly all strongly sex‐associated (large residual) SNPs clustered in a central area between 33.0 and 48.7 cM, with medium‐residual (marginally sex‐associated) SNPs also distributed up to the end of the linkage group from 48.7 to 60.2 cM. As theory predicts that the most strongly sex‐associated loci cluster around the sex‐determining locus, this suggests that a sex‐determining locus in *L. saxatilis* is located in the region from 33.0 to 48.7 cM (LGC12.2 and LGC12.3; see below). Indeed, a strong QTL for sex (LOD = 26, *P* < 0.001) in *L. saxatilis* has recently been identified on LG12 (Koch et al. 2021) and is located in the central region of sex‐associated SNPs. Thus, our data support the presence of a sex‐determining region on LG12 and show that it is a female‐heterogametic system, but only in the Crab ecotype.

### SEX‐DETERMINATION IN THE WAVE ECOTYPE

All evidence for a female‐heterogametic sex determination system was found in the Crab ecotype only, leaving the mechanism for sex determination in the Wave ecotype unknown. With such close proximity to the Crab ecotype, there is likely to be some genetic component of sex determination in Wave; this may or may not involve the same sex‐determining locus as in Crab. Any weaker patterns in Wave may have been masked by the strong Crab pattern. Therefore, the comparison of heterozygosity between sexes was repeated for all linkage groups with Crab and Wave individuals separated. Results were more variable in Wave, probably due to the lower sample sizes of Wave males and females, and displacement of clines into the Wave habitat (Westram et al. 2018) (Fig. S1). One linkage group, LG5, showed a likely signal (Figs. 2B, S1) with some female bias in heterozygosity, although much weaker than that seen on LG12 in Crab. About 60% of the most sex‐associated SNPs (1% most negative residuals) were located on LG5, whereas no other linkage group held more than 6% of these SNPs (Fig. S1). SNPs with strongly negative residuals (female heterozygosity > male heterozygosity) were spread across much of LG5 (Fig. 2D). One possible explanation for this pattern is a young ZW system, with less differentiation than for LG12 in Crab. In this study, we focus on the LG12 sex‐determination system; future analysis is needed to determine any potential role of LG5 in Wave.

### PUTATIVE INVERSIONS ON LG12

Inversions are often found on sex chromosomes. They are hypothesized to be a key mechanism in the suppression of recombination during the evolution of sex chromosomes (Lahn and Page 1999; Wang et al. 2012; Natri et al. 2019), but they may evolve later following recombination suppression by other means. Whether a cause or consequence, inversions are expected in sex‐determining regions.

**Figure 3:**
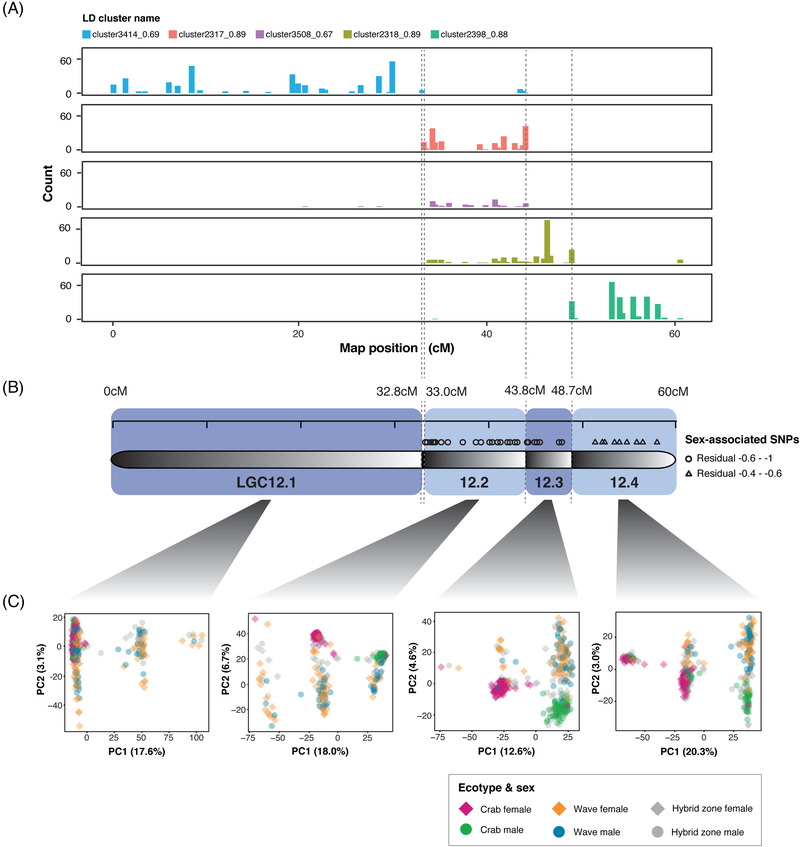
(A) The distribution of SNPs along LG12 in each of the five LD clusters of interest, and how these correspond to (B) four putative inversion regions on LG12 (illustrated by black‐white gradients) and the distribution of sex‐associated SNPS from Figure 1. (C) PC1 versus PC2 from PCAs (scaled and centered) of SNPs in the four regions of LG12 covered by the LD clusters of interest. PCAs were carried out for all individuals (of both sexes and ecotypes) together.

Therefore, LD and PCA were carried out to test for the presence of sex‐specific inversions on LG12 that cover the region of sex‐associated SNPs. Five outlier clusters of SNPs were identified, using LDna for females, as regions of interest for downstream analysis (see *Results* in the Supporting Information; Fig. 3A; Table S1). SNPs in each of the five clusters were distributed in distinct regions of LG12 (Fig. 3A,B). Two clusters covering the first and last parts of LG12 match the positions of the inversions LGC12.1 and LGC12.2, respectively, from Faria et al. (2019). The other three clusters overlap and cover the central region of LG12 between the two described inversions (Fig. 3A,B), suggesting previously undiscovered putative inversions that span the central region of LG12.

PCA was carried out on the three regions of LG12, both separately for males and females and with the sexes together. Six distinct clusters were present in the PCA of the central region, a pattern consistent with two neighboring inversions in LD with one another. This was corroborated by examining genotypes of individuals across LG12 (Figs. 3B, S2A,B, S3; see *Methods* and *Results* in the Supporting Information for a detailed explanation). This central region was therefore split into two according to the SNP and genotype distributions, and PCAs of these subregions each gave three distinct groups along PC1 with either no or very rare intermediate individuals (Figs. 3C, S3). The overlapping clusters observed in LDna (Fig. 3A) were likely due to LD between these two putative inversions. For the first and last region, separate PCAs of females and males (Fig. S3) revealed the expected three distinct groups, consistent with polymorphism of LGC12.1 and LGC12.2 (as detected by Faria et al. 2019) in both sexes. For each of the four putative inversion regions, snails from all locations across the transect were present within the same three clusters indicating that the arrangements are shared between ecotypes. PCA using both sexes together for each region showed that males and females also fell into the same three distinct groups (one group is very small for region 43.8–48.7 cM, where one putative homozygote is rare) (Fig. 3C): that is, sexes also share arrangements of the putative inversions. Arrangement frequencies are examined in the next section.

The LD and PCA supported the presence of four putative polymorphic inversions on LG12 (Fig. 3B). From this point on, the putative inversions will be referred to simply as inversions, for brevity, and named LGC12.1 (the same as LGC12.1 from Faria et al. [2019]), LGC12.2, LGC12.3, and LGC12.4 to maintain the inversion naming system used in Faria et al. (2019). LGC12.2 of Faria et al. (2019) is renamed to LGC12.4 so that names are sequential along LG12.

**Figure 4:**
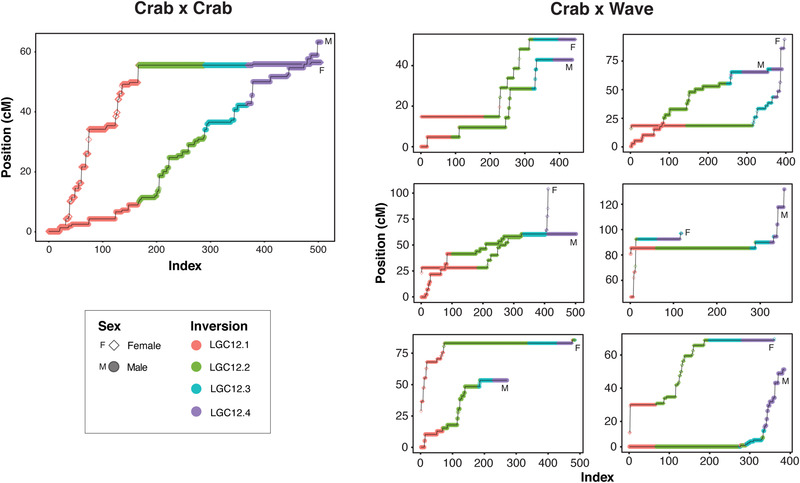
Male‐ and female‐specific genetic maps of LG12 from a Crab × Crab family, and six Crab × Wave F2 families. For the Crab × Crab map, only markers informative in both sexes were used. In the Crab × Wave maps, markers informative in females only were used for the female map and markers informative in males only were used for the male map. In all panels, markers were numbered in order according to their position on LG12 (Index). Markers were colored by putative inversion region, with assignment based on their positions relative to the outermost map positions of markers confidently assigned to each inversion. Markers that could not be assigned to an inversion were removed. Horizontal lines (i.e., no change in map position between successive markers) indicate an absence of recombination.

Recombination is expected to be suppressed in individuals heterozygous for inversion arrangements and, therefore, genetic maps can help to confirm the presence of inversions. Maps for each sex from a Crab × Crab family (Westram et al. 2018) and a series of Crab × Wave families (Koch et al. 2021) further supported the presence of inversions in the genomic locations described (Fig. 4). In the Crab × Crab map, both parents showed normal recombination in the first part of LG12, whereas recombination was absent in the female parent in the second half of the linkage group where sex‐associated SNPs are found. This is consistent with the female parent being heterozygous for inversions LGC12.2, LGC12.3, and LGC12.4. In the Crab × Wave families, each parent showed a different pattern of recombination, consistent with different combinations of heterozygous inversions in these hybrid individuals. In each case, blocks of low recombination corresponded to one or more of the four inversions (Fig. 4). These maps support the interpretation that Wave males can have any genotype for any of the four putative inversions, unlike Crab males that are nearly always homozygous for LGC12.2 and LGC12.3.

The fragmented *L. saxatilis* genome assembly and the capture sequencing approach used here preclude formal confirmation that regions of suppressed recombination are caused by inversions. However, other possible mechanisms of recombination suppression, in sex chromosome evolution and otherwise, such as transposable elements, heterochromatinization, methylation, and epigenetic effects (Ironside 2010; Furman et al. 2020) are unlikely to produce the specific patterns we observe in this study (namely, the clustering of high LD SNPs in specific regions, the identification of three genetically distinct clusters of individuals by PCA, and the genotype‐specific recombination suppression in experimental crosses) (Kemppainen et al. 2015; Faria et al. 2019).

### ECOTYPE DIFFERENCES IN SEX‐INVERSION ASSOCIATIONS

**Figure 5:**
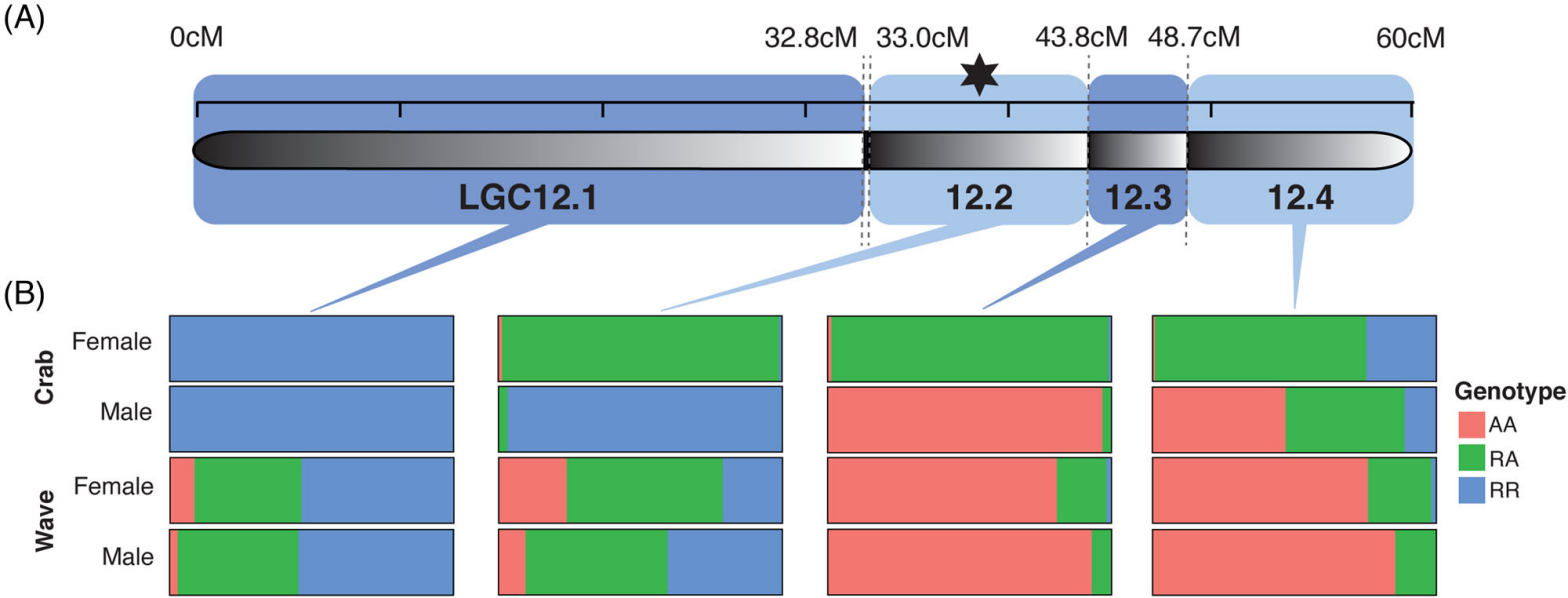
The location of the four putative inversions on LG12 and the proportions of inversion genotypes for each sex and ecotype group. Arrangements are labeled R (reference: the arrangement more frequent in Crab than Wave in females) and A (alternate); thus, RR and AA are the two homozygote groups and RA is the heterozygote group. Black star indicates the approximate position of the QTL for sex (Koch et al. 2021).

Associations among genotypes at putative inversions, and between putative inversions and sex, were quantified in both ecotypes. Inversions that are involved in sex chromosome evolution are expected either to contain the sex‐determining locus or be in LD with it. Therefore, we predicted that LGC12.2, LGC12.3, and LGC12.4 would show significant association with sex and with each other in the Crab ecotype, but not the Wave ecotype. In the Crab ecotype, inadequate polymorphism meant associations could not be calculated: all individuals were fixed for one arrangement at LGC12.1, almost all females were heterozygous at LGC12.2 and LGC12.3, and almost all males were homozygous for one arrangement at LGC12.2 and LGC12.3 (Fig. 5). In the Wave ecotype, correlations between inversions were generally low and seven of the 12 pairwise comparisons were nonsignificant (Table 1). Significant correlations were present between LGC12.1 and LGC12.2 and between LGC12.3 and LGC12.4 in both sexes in Wave (Table 1). These relationships fulfilled our predictions (Fig. 5; Table 1): within Crab, LGC12.2 and LGC12.3 were significantly correlated with sex, LGC12.4 was less strongly correlated, although the relationship was still highly significant, and in Wave correlations with sex were weak, although the order between them was the same (i.e. LGC12.2 showed the strongest, but only marginally significant, association; Table 1). A small number of Crab‐like individuals present in the Wave habitat may have influenced these correlations (consistent with genome‐wide clinal patterns seen in Westram et al. 2018).

**Table 1:** Correlation (*r*_2_) and significance of association of genotypes at pairs of inversions for males and females of the Wave ecotype. Correlation and significance of association of inversion genotype with sex is also given in the final column for the Crab and Wave ecotypes

	LGC12.2		LGC12.3		LGC12.4		Sex	
	F	M	F	M	F	M	C	W
LGC12.1	**0.3969** (0.000133)	**0.5929** (4.82 × 10^−5^)	0.0064 (0.784)	0.0100 (0.352)	0.0100 (0.736)	0.0004 (0.499)	N/A	0.0036 (0.420)
LGC12.2			0.1936 (0.0447)	0.0784 (0.387)	0.1225 (0.176)	0.0081 (0.745)	**0.9025** (4.24 × 10^−30^)	0.0625 (0.0428)
LGC12.3					**0.7921** (2.30 × 10^−10^)	**0.4624** (0.000427)	**0.9025** (4.24 × 10^−30^)	0.0289 (0.220)
LGC12.4							**0.2116** (1.33 × 10^−10^)	0.0169 (0.393)

Abbreviations: C = Crab; F = female; M = male; W = Wave.

Correlations with a significance of p < 0.05 were highlighted in bold.

Differences in arrangement frequency along the transect were quantified for males and females as a proxy for divergent selection on the arrangements between the ecotypes and to test how this differed between the sexes. If a difference in sex determination system between ecotypes is maintained by selection despite gene flow, inversions associated with sex (LGC12.2 and LGC12.3) will show clines in frequency between environments that differ between the sexes. Inversions not associated with sex (LGC12.1) may show clines, as previously shown in Faria et al. (2019), but these are not expected to differ between the sexes. The expectation for LGC12.4 is equivocal because of its partial association with sex.

**Figure 6:**
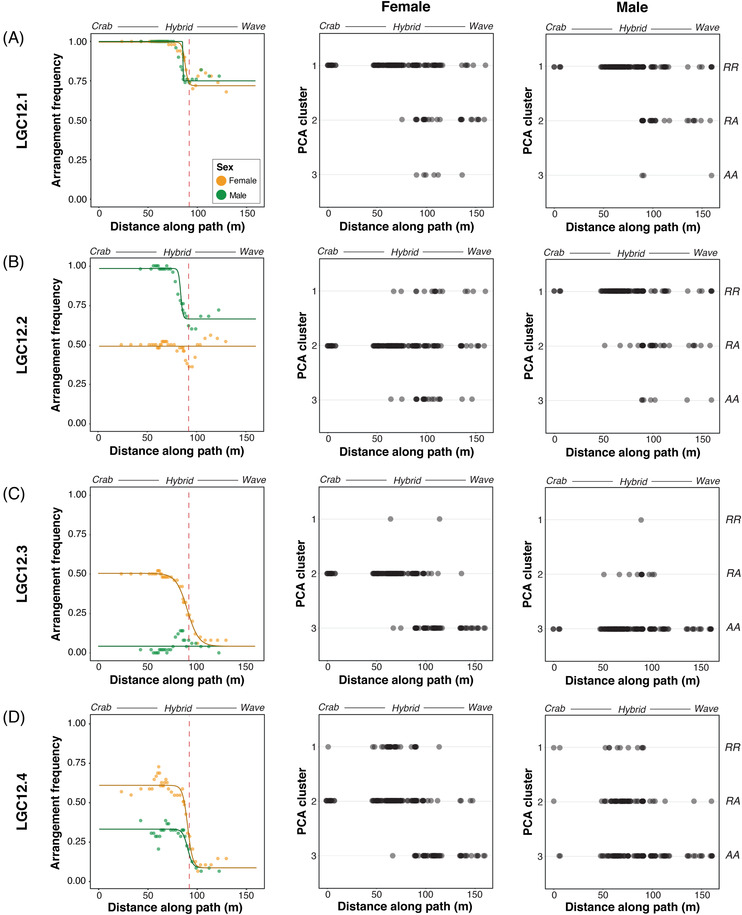
*Left‐hand panels*: Frequency of R arrangement in windows of snails across the transect, and best fitting cline models of R arrangement frequency, in males and females for (A) LGC12.1, (B) LGC12.2, (C) LGC12.3, and (D) LGC12.4. Red dashed line shows the mean cline center for nonneutral SNPs (91.8 m) from Westram et al. (2018). Labels above each panel show the direction of transect (Crab‐Hybrid zone‐Wave); these labels are illustrative only because phenotypic and genetic clines vary in width and position. The best fitting cline models for each inversion (and for each sex separately where the best fitting model differed between sexes) were as follows: LGC12.1—full model; LGC12.2 females—null model, males—full model; LGC12.3—Wave‐constrained; and LGC12.4—Wave‐constrained. *Center and right‐hand panels*: Distribution along the transect of individuals in each PC1 cluster for (A) LGC12.1, (B) LGC12.2, (C) LGC12.3, and (D) LGC12.4 for females (*center*) and males (*right*). PCA cluster 1 corresponds to R arrangement homozygotes; inversion genotypes (RR, RA, AA) are noted on the right‐hand side for ease.

Clines in arrangement frequency between ecotypes were detectable for all inversions for one or both sexes, indicating a role of divergent selection (Fig. 6; Tables S2 and S3). No inversion showed a sex difference in cline center or width, and all fitted cline centers were close to the mean position of nonneutral clines from throughout the genome reported by Westram et al. (2018); the same environmental transition is likely to be driving selection on LG12 as the rest of the genome. As predicted, males and females showed little difference in arrangement frequency in either ecotype in LGC12.1, but arrangement frequencies differed between males and females in the Crab ecotype for the other three inversions. In addition, a small sex difference in arrangement frequencies may be present in the Wave ecotype for LGC12.2 (the “Wave‐constrained” model was marginally worse than the “constrained” cline model). A clear shift in genotype frequencies for LGC12.2 was visible in females from Crab to Wave, from all heterozygotes to approximate Hardy‐Weinberg proportions, despite there being no cline in arrangement frequency (Fig. 6B).

Sex differences in SNP heterozygosity and putative inversion genotypes were found only in the Crab ecotype, whereas no sex differences could be seen among Wave individuals. Transitions in arrangement and genotype frequencies occurred over a short distance (0–23 m; Table S2). This indicates strong differential selection on the sex‐determining region (LGC12.2 and LGC12.3) because ecotypes are connected by gene flow across the hybrid zone (Westram et al. 2018). Our PCA analysis confirmed that the three inversions (regions with suppressed recombination in heterozygotes) that are sex associated in Crab were also present in Wave. However, as suggested by the lack of sex‐association in Wave in the heterozygosity analyses, there is no evidence that the Wave ecotype follows the same sex‐determining system as we have found in Crab. The QTL for sex in the Crab × Wave F2 crosses (Koch et al. 2021) was produced by alleles derived from the Crab parents; Crab females and Wave males were used as parents for the crosses, so any female‐specific sex‐determining alleles would be derived from the Crab ecotype.

Both arrangements of LGC12.2, the primary sex‐determining region in Crab, were present in both sexes at an intermediate frequency in Wave. Wave females showed all three putative inversion genotypes in approximately Hardy‐Weinberg proportions (Fig. 5). Wave males similarly showed all three putative inversion genotypes, with a slightly higher frequency of 0.7 of the R arrangement (defined as the one more frequent in Crab than Wave in females, or in males if female frequency does not change). If the sex‐determining locus is the same in Crab and Wave, for the three putative inversion genotypes to be present in both sexes, haplotypes of both the arrangements must exist with each of the alleles at the sex‐determining locus to remove the sex‐inversion association. In contrast, in Crab the female‐specific allele at the sex‐determining locus must be present on the A background only. This may be a shared haplotype with the Wave ecotype. Similarly, the other (male) allele at the sex‐determining locus on the R arrangement in Crab may be shared across the transect into Wave. The lack of elevated divergence between Crab and Wave for any arrangement in either sex supports this idea (Fig. S4B).

The R arrangement of LGC12.3 was present at only a low frequency in the Wave ecotype (around 0.1), with the majority of Wave individuals of both sexes being homozygous for the A arrangement. In males, the R arrangement was present only near the hybrid zone, whereas Crab females were heterozygous. The presence of the R arrangement predominantly in Crab females suggests the origin of the R arrangement in this group and its failure to spread into the Wave habitat. This is consistent with the evolution of LGC12.3 as a second stratum of a female heterogametic sex chromosome in Crab. However, the presence of male heterozygotes and RR homozygotes of both sexes in the hybrid zone would indicate that rare recombination events occur in hybrids: If the putative inversions arose sequentially (as proposed below), recombination must have occurred between the close breakpoints of LGC12.2 and LGC12.3 to associate the R arrangement of LGC12.3 with an arrangement lacking the female sex‐determining allele on LGC12.2. Individuals with these genotypes are limited to the hybrid zone, however, implying that the genotypes are not fit enough to spread into either the Crab or Wave environment.

Sex differences in genotype and arrangement frequencies in both ecotypes were less distinct for LGC12.4. Similar to LGC12.3, the R arrangement was present at a low frequency in both sexes in the Wave ecotype and R homozygotes were rarely seen away from the hybrid zone. However, the strong genotypic differences between the sexes in LGC12.2 and LGC12.3 in Crab were not present for LGC12.4. The R arrangement differed in frequency slightly between males and females (0.3 and 0.6, respectively), but all three genotypes were seen in both sexes. Associations between LGC12.4 and the other inversions were generally rather low, suggesting one of two things: Either, this inversion did not evolve for reasons relating to sex and is just in LD with LGC12.3 due to their shared or close breakpoints, resulting in small sex differences in frequency. Or, any sex‐specific benefits of the association of an LGC12.4 arrangement with sex (and therefore with the other inversions) are only just emerging, so the beneficial combination of arrangements among inversions has not yet spread. For example, if sexual antagonism was playing a role in this system, this could occur with a recent change so that a locus in LGC12.4 becomes sexually antagonistic, creating selection for association of a particular haplotype of a preexisting inversion with sex.

### DIVERSITY AND DIVERGENCE OF PUTATIVE INVERSION ARRANGEMENTS

Genetic diversity (π) for each arrangement can be compared for an insight into which inversion arrangement is derived. Young inversions are expected to show low diversity in the derived arrangement compared to the ancestral, whereas the derived arrangement of older inversions is expected to have accumulated diversity over time, reducing the difference between the two arrangements (Andolfatto et al. 2001; White et al. 2009). At the same time, divergence (*d*_XY_) between arrangements is expected to increase as they age.

**Figure 7:**
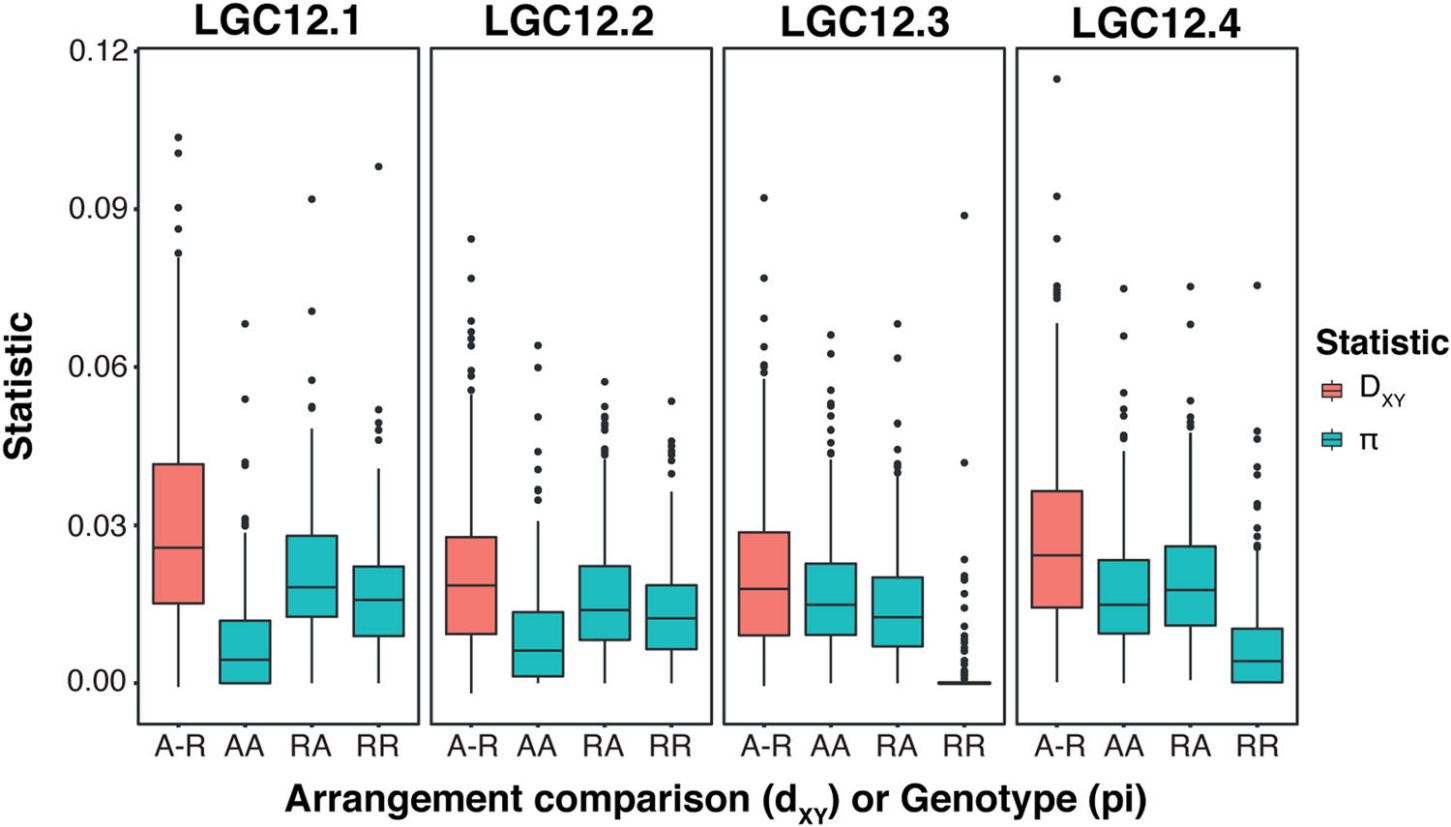
π per contig of each inversion genotype and *d*_XY_ between inversion arrangements for the four inversions.

The arrangement with lower π was identified through comparison of homokaryotypes for each arrangement. In the case of LGC12.3, where one homokaryotype was extremely rare, the heterokaryotype showed lower π than the abundant homokaryotype, implying a lower π in the rare than abundant homokaryotype. The R arrangement had lower π, and was inferred to be derived, for LGC12.3 and LGC12.4, whereas the A arrangement had lower π for LGC12.1 and LGC12.2 (Fig. 7). Models confirmed that genotype significantly affected π for each of the four inversions (Tables S4–S6).

For the three inversions in the sex‐associated chromosomal region, *d*_XY_ between arrangements was similar with a possible slight elevation in LGC12.4 (Fig. 7). LGC12.2 showed the smallest difference in π between the two homokaryotypes, although estimates were again relatively similar between inversions (Fig. 7). These *d*_XY_ and π estimates suggest that LGC12.2 may be the oldest of the sex‐associated putative inversions. Estimates of *d*_XY_ did not reveal any marked differences between sexes or ecotypes for any inversion (Fig. S4B), suggesting that no arrangement is diverging more rapidly than the others between sexes or ecotypes. However, there were differences in π between ecotypes and sexes for the sex‐associated inversions (Tables S5 and S6), although estimates were generally much smaller than the genotype effect. The Crab ecotype showed reduced π compared to Wave in models for all three sex‐associated inversions. Females showed reduced π compared to males in LGC12.3 and LGC12.4, but showed slightly higher π than males in LGC12.2. Significant effects of ecotype and sex on π were likely produced by the differing frequencies of arrangements among sexes and ecotypes, but may imply that certain haplotypes of an arrangement are not shared between groups. Such differences can also be seen in the PCAs, where ecotypes are partly differentiated within each PC1 cluster (Fig. 3C).

### SEX CHROMOSOME STRATA IN THE CRAB ECOTYPE

The association of inversions with regions of sex‐associated SNPs aligns with theory on sex chromosome strata. The putatively derived arrangements of LGC12.2 and LGC12.3 are restricted to the heterozygous females in Crab, whereas males only exhibit the ancestral arrangement. These genotypes are expected if inversions are selected for recombination suppression in the heterogametic sex. The oldest stratum is expected to contain the sex‐determining locus and LGC12.2, likely to be the oldest inversion on the basis of diversity and divergence, contains the sex QTL. Diversity estimates are less clear in distinguishing the age of LGC12.3 and LGC12.4. A greater difference in diversity between arrangements is visible in LGC12.3, but the comparison is unreliable due to the low derived arrangement frequency. The strong association of LGC12.3 with sex is a better indicator that it is older than LGC12.4 and evolved second. LGC12.4 shows much smaller sex differences in arrangement and SNP genotype frequencies, suggesting it may be the youngest stratum that has not yet spread throughout the population. However, this pattern may also be produced by differing amounts of recombination between putative inversions. More recombination between LGC12.3 and LGC12.4 than between LGC12.2 and LGC12.3 would result in a weaker association of inversion genotype with sex for LGC12.4. Conversely, LGC12.4 may only show sex association at all due to chance buildup of LD between itself and LGC12.3 if opposed only by low recombination. Although recombination was not observed in the region of sex‐linked putative inversions in the Crab × Crab recombination map, the size of the family used means that a map distance of around 1 cM between LGC12.3 and LGC12.4 remains plausible. Neither the genetic maps nor the genome assembly currently available make it possible to be certain of the relative positions of breakpoints for the four putative inversions.

### SCENARIOS FOR SEX‐ AND ECOTYPE‐SPECIFIC PATTERNS OF SELECTION

Here, we speculate about possible evolutionary histories that may have produced the patterns of inversion polymorphism we observe. Cline analysis revealed distinct changes in arrangement frequency between the ecotypes for all four inversions (Figs. 5, 6), indicating a role of divergent selection between habitats. Previous evidence for adaptive trait QTL and outlier SNPs on LG12 (Morales et al. 2019; Koch et al. 2021; Westram et al. 2021) supports this. LG12 contributes strongly to phenotypic variation in shape, aperture, and shell length (Koch et al. 2021), suggesting the presence of alleles under strong habitat‐specific selection. Our analyses here also highlight a role for sex‐specific selection in the Crab ecotype. At the moment, it is not clear whether the spread of the inversions was first promoted by divergent selection between ecotypes or by their role in sex chromosome evolution.

Several potential drivers for recombination suppression in the evolution of sex chromosomes have been discussed, including genetic drift, heterozygote advantage, and meiotic drive as well as sexual antagonism, but evidence distinguishing them remains scarce (Ironside 2010; Charlesworth 2017; Ponnikas et al. 2018). As the drift hypothesis requires small population sizes and heterozygote advantage is favored in inbreeding populations, neither mechanism seems likely to explain the strong ecotype differences we observe. Sexually antagonistic selection remains the predominant theory for the evolution of sex chromosomes (Fisher 1931; Rice 1996) and it seems plausible for *L. saxatilis* because the effects of a trait on male and female fitness can depend on the local environment (Connallon & Clark 2014; Connallon 2015), potentially resulting in environment‐dependent sexual antagonism. However, note that population differences in sex chromosomes can occur without invoking the need for varying sexual conflict (Bergero & Charlesworth 2019). If it is assumed that sexual antagonism did indeed play a role in the evolution of this young sex‐determining region, at least two scenarios can be considered for the evolution of the LG12 putative inversions. In one, LGC12.2 first evolved in Crab females due to the presence of a locus with sexually antagonistic effects close to the sex‐determining locus. The sexual dimorphism selected for in Crab was disadvantageous in Wave. The derived arrangement spread into Wave, for reasons unknown, and lost its association with sex through rare recombination events during interbreeding in the hybrid zone, which placed the Z allele at the sex‐determining locus onto the derived background. Another scenario is possible where LGC12.2 first appeared in the Wave ecotype and spread because it enhanced local adaptation. Recombination allowed both Z and W alleles at the sex‐determining locus to be present on the derived arrangement. Sexual antagonism was not the driver of the evolution of the putative inversion in this case; however, it remains necessary to explain the spread into Crab of only the derived arrangement carrying the female‐specific (W) allele at the sex‐determining locus. Both scenarios require disparate selection on males and females between the two ecotypes; some aspect of the Crab environment creates differential fitness effects of a trait for males and females, but this does not occur in the Wave habitat. One potential example of such a trait is size; size dimorphism between the sexes is more pronounced in Crab than Wave (Perini et al. 2020). In Crab, males mature early to enable mating as early as possible, whereas females need to create space for as many embryos as possible and therefore grow larger and mature later. However, in Wave a large size is selected against in both males and females as individuals must fit into small crevices for protection from waves.

These scenarios also assume that the same sex‐determining locus is present in both Crab and Wave. Whether this is the case is unknown. If Wave does not share the LG12 sex‐determining locus, many more possibilities for different scenarios of selection are possible. For example, the putative inversion may have arisen and spread among the two ecotypes due to locally adaptive effects, as with other inversions in *L. saxatilis*. Subsequent, sexual antagonism in Crab could have led to a new female‐determining allele arising within the derived arrangement in Crab, spreading to fixation on that arrangement and reducing its male‐specific fitness such that it was lost and the ancestral arrangement was fixed in males. Again, the lack of sexual antagonism prevented the spread of this haplotype into Wave. This scenario has the advantage that it does not rely on unexplained spread of an arrangement between ecotypes and rare recombination to alter the relationship between the sex‐determining alleles and putative inversion arrangements. It predicts that sex is determined by a different locus in Wave and our analyses suggest that this locus could be on LG5.

A similar pattern of selection is required to explain the evolution of LGC12.3. The strong association with sex and predominant presence of the derived arrangement in Crab females only supports a role of sexually antagonistic selection in Crab to create a second stratum of a sex‐determining region. Again, a strong barrier to spread of the derived arrangement into Wave must exist because most Wave individuals are homozygous for the ancestral arrangement. Capture of locally adaptive loci may be involved in the maintenance of this barrier; however, the strong sex association means it is improbable that divergent natural selection alone would produce the observed ecotype differences. The clinal variation in arrangements of LGC12.4 but weak sex association gives weight to the possibility that this inversion is predominantly involved in ecotypic rather than sex differentiation.

The disparity between the ecotypes in the emergence of a sex‐determining region is striking. Populations are only a few meters apart and readily interbreed in the hybrid zone. There is no evidence for substantial periods of allopatric divergence (Butlin et al. 2014). The distinct barrier to the spread of the sex‐determining region from the Crab ecotype into Wave indicates that there must be a difference in the selective regime acting on the two ecotypes. Although clearly very complex and not yet fully understood, this undoubtedly must involve sex‐specific selection as well as the divergent natural selection previously characterized in *L. saxatilis*. Further analysis of this system, including additional hybrid zones across Europe, will aid understanding of this intricate pattern and is likely to give new insights into both local adaptation and sex chromosome evolution.

## AUTHOR CONTRIBUTIONS

RKB, KJ, and AMW designed the project and collected the data. KEH led the data analysis with input from RF, ELK, and SS. KEH wrote the manuscript with input from all authors.

## CONFLICT OF INTEREST

The authors declare no conflict of interest.

## DATA ARCHIVING

No new data were generated for these analyses. See Westram et al. (2018) and Koch et al. (2021) for access to previously published data. Analysis scripts, and data files saved at intermediate steps for simplicity, are available on GitHub at https://github.com/katiehearn/Littorina_sex_ANG.

## Supporting information

Supporting information

Supporting Figure S1

Supporting Figure S2

Supporting Figure S3

Supporting Figure S4
