# Supplementary material for: Differing associations between sex determination and sex‐linked inversions in two ecotypes of Littorina saxatilis
Source: Evol Lett. 2022 Aug 12;6(5):358–74. doi: 10.1002/evl3.295 (PMC9554762; doi:10.1002/evl3.295)
Supplement: Supplementary file 1 — Supporting information [file EVL3-6-358-s003.docx]

**Supporting Methods**

*Inversion detection using LDna and PCA*

The two main parameters in LDna, |E|_min_ and φ, were manipulated to investigate the detection of clusters. |E|_min_ represents the minimum number of edges required for a cluster to be an outlier, where edges are connections between pairs of SNPs that are in LD above a threshold. This is correlated to some extent with the number of SNPs in a cluster. The other parameter, φ, relates to the minimum LD threshold for a cluster to be considered an outlier; it compares the median intracluster pairwise LD to intercluster LD. A greater value of φ indicates that the cluster ‘stands out’ more against the background LD. Various combinations of the two parameters were tested, with |E|_min_ between 30 and 4000 and φ between 1 and 10, and the output tables of putative outlier clusters saved for further investigation.

Low values of the two parameters are less ‘stringent’ so many more clusters were retained in the output. Clusters with few edges are more likely to be small clusters of SNPs in close physical linkage. Clusters with a median pairwise LD below 0.3 were excluded since loci in inversions are expected to be under high LD. The distributions of SNPs in clusters were checked, as SNPs in clusters representing inversions are expected to be located in one region of the linkage group rather than scattered. The identities of SNPs in clusters were also checked as one large cluster detected under a certain combination of parameters was often detected as a few smaller clusters under different parameter combinations.

The set of clusters that was repeatedly detected under numerous different parameter combinations, and passed the above checks, was retained for downstream principal component analysis. The first two principal components were examined for the distinctive three groups on PC1 that indicate individuals that are homokaryotypic for each inversion arrangement, or heterokaryotypic. Distinct clusters on PC1 without intermediates are produced due to the lack of recombination between alternate arrangements, which allows allele frequencies to diverge between arrangements for many SNPs. The central cluster represents heterokaryotypes since they hold intermediate allele frequencies. Individuals were manually assigned a genotype (hom1, het, hom2) based on their PC1 grouping; the small numbers of individuals that were not clearly part of a cluster were assigned an *N/A* value.

*Testing the effect of genotype, sex, and ecotype on π*

Any group with fewer than two individuals was excluded (see Supporting Table 4 for the number of snails in each group). Groups were also reordered to ensure the group taken as the intercept by the model was not empty; the Wave female heterozygote group (F_RA_W) was used as the intercept for all inversions. Values were log-transformed to give normally distributed data before models were fitted. Map position was included as a random effect as contigs at the same map position are not independent. Random slopes and intercepts were included in the model. The full model (which includes all fixed effects and interactions) was used as the global model in the MuMIn dredge() function to determine the best fitting model. This function tested all combinations of fixed effects and interactions. Only models with a ΔAIC<2 compared to the best fitting model were retained. For inversions where only one model was retained, estimates were extracted. Where more than one model was retained for a putative inversion, models were averaged using the MuMIn function model.avg() before the model-weighted average estimates were extracted.

**Supporting Results**

*Inversion detection*

Many ‘outlier clusters’ were detected in the LDna investigation (Supporting Table 1). Only five clusters appeared repeatedly at all parameter combinations, while others only appeared at certain parameter values or did not meet minimum threshold requirements or other checks. This was especially notable at high values of |E|_min_ and φ (end of Supporting Table 1), where the outputs consisted mostly of the five clusters. Small clusters appearing at lower parameter values were mostly just small subsets of the five clusters. As a result, these five clusters were selected for continued analysis.

The distribution of SNPs in the clusters along LG12 was plotted (Figure 3b). One cluster covered the first half of the linkage group and another the end section, but the other three clusters had overlapping distributions in the central region of LG12. The three clusters all started from the same point on LG12, but one cluster covered a longer region of the linkage group than the other two clusters. A PCA of the central region covered by the three clusters revealed 6 groups separated by PC1 and PC2 (Supporting Figure 2a). This is indicative of two inversions in LD with each other, as each cluster represents one of the six combinations possible of the three genotypes at the two inversions, overlapping or in LD. This was supported by examining haplotypes of individuals along LG12 (Supporting Figure 2b); the central region covered by the three LDna clusters was clearly split into two regions with differing haplotypes. The map position where the central region haplotypes split was the same as the point where two of the three central LDna clusters end. It was therefore clear that the three LDna clusters in the centre of LG12 represented two adjacent inversions. Non-random association of genotypes for the two inversions probably explains the generation of overlapping LDna clusters. From this point on, these two regions were used for analysis in addition to the two regions represented by the LDna clusters at the start and end of LG12.

**Supporting Tables and Figure Legends**

**Supporting Table 1.** Full LDna cluster outputs for varying combinations of the input parameters phi (φ) and minimum number of edges (|E|_min_) (columns one and two). Outputs are ordered in ascending values of |E|_min_ and phi. The five clusters of interest are shaded in grey every time they appear.

| Edges | Phi | Cluster name | Merge.at | nLoci | nE | lambda | Median.LD | MAD.LD |
| --- | --- | --- | --- | --- | --- | --- | --- | --- |
| 30 | 1 | 1509_0.96 | 0.95 | 133 | 5085 | 3.99 | 0.964 | 0.024 |
| 30 | 1 | 1837_0.94 | 0.93 | 207 | 10310 | 4.14 | 0.932 | 0.0416 |
| 30 | 1 | 2043_0.92 | 0.91 | 124 | 3950 | 8.68 | 0.921 | 0.0534 |
| 30 | 1 | 2046_0.92 | 0.91 | 183 | 4899 | 3.66 | 0.868 | 0.0648 |
| 30 | 1 | 2329_0.89 | 0.86 | 10 | 38 | 4.9 | 0.977 | 0.0121 |
| 30 | 1 | 2406_0.88 | 0.86 | 31 | 194 | 8.37 | 0.85 | 0.14 |
| 30 | 1 | 2640_0.85 | 0.6 | 23 | 232 | 20.47 | 0.97 | 0.0148 |
| 30 | 1 | 2849_0.81 | 0.6 | 12 | 34 | 7.56 | 0.807 | 0.074 |
| 30 | 1 | 2927_0.79 | 0.74 | 19 | 160 | 3.8 | 0.978 | 0.0169 |
| 30 | 1 | 2943_0.79 | 0.52 | 18 | 107 | 12.78 | 0.921 | 0.06 |
| 30 | 1 | 3026_0.77 | 0.73 | 36 | 189 | 4.32 | 0.673 | 0.127 |
| 30 | 1 | 3217_0.73 | 0.71 | 30 | 165 | 4.5 | 0.659 | 0.116 |
| 30 | 1 | 3228_0.73 | 0.66 | 16 | 58 | 7.44 | 0.696 | 0.178 |
| 30 | 1 | 3243_0.73 | 0.54 | 13 | 39 | 9.035 | 0.702 | 0.105 |
| 30 | 1 | 3334_0.71 | 0.64 | 30 | 146 | 6.9 | 0.611 | 0.151 |
| 30 | 1 | 3517_0.67 | 0.59 | 16 | 62 | 3.92 | 0.704 | 0.162 |
| 30 | 1 | 3596_0.65 | 0.62 | 22 | 106 | 11.44 | 0.62 | 0.15 |
| 30 | 1 | 3608_0.65 | 0.48 | 11 | 32 | 8.36 | 0.768 | 0.182 |
| 30 | 1 | 3653_0.64 | 0.49 | 10 | 37 | 9.7 | 0.98 | 0.0129 |
| 30 | 1 | 3671_0.63 | 0.62 | 36 | 124 | 10.08 | 0.423 | 0.141 |
| 30 | 1 | 3674_0.63 | 0.62 | 11 | 32 | 4.235 | 0.745 | 0.17 |
| 30 | 1 | 3693_0.63 | 0.51 | 13 | 39 | 7.605 | 0.618 | 0.162 |
| 30 | 1 | 3736_0.62 | 0.48 | 10 | 37 | 6.7 | 0.936 | 0.0508 |
| 30 | 1 | 3767_0.61 | 0.59 | 17 | 53 | 6.46 | 0.527 | 0.226 |
| 30 | 1 | 3852_0.59 | 0.58 | 26 | 96 | 10.92 | 0.445 | 0.179 |
| 30 | 1 | 3888_0.59 | 0.37 | 18 | 81 | 11.88 | 0.674 | 0.112 |
| 30 | 1 | 3942_0.57 | 0.56 | 24 | 91 | 11.4 | 0.487 | 0.189 |
| 30 | 1 | 3944_0.57 | 0.56 | 19 | 55 | 6.46 | 0.38 | 0.17 |
| 30 | 1 | 4010_0.56 | 0.51 | 16 | 48 | 5.12 | 0.506 | 0.126 |
| 30 | 1 | 4050_0.55 | 0.49 | 17 | 36 | 5.27 | 0.362 | 0.0957 |
| 30 | 1 | 4058_0.55 | 0.46 | 12 | 35 | 4.26 | 0.555 | 0.322 |
| 30 | 1 | 4074_0.55 | 0.29 | 11 | 44 | 7.59 | 0.702 | 0.153 |
| 30 | 1 | 4105_0.54 | 0.49 | 15 | 71 | 4.65 | 0.72 | 0.204 |
| 30 | 1 | 4127_0.54 | 0.29 | 15 | 63 | 9 | 0.614 | 0.168 |
| 30 | 1 | 4146_0.53 | 0.51 | 31 | 151 | 4.34 | 0.409 | 0.121 |
| 30 | 1 | 4195_0.52 | 0.49 | 15 | 35 | 4.8 | 0.348 | 0.175 |
| 30 | 1 | 4241_0.51 | 0.47 | 14 | 57 | 9.1 | 0.657 | 0.203 |
| 30 | 1 | 4253_0.51 | 0.43 | 18 | 37 | 3.96 | 0.233 | 0.149 |
| 30 | 1 | 4257_0.51 | 0.42 | 17 | 97 | 11.05 | 0.649 | 0.299 |
| 30 | 1 | 4266_0.51 | 0.33 | 10 | 30 | 7.3 | 0.732 | 0.245 |
| 30 | 1 | 4274_0.5 | 0.49 | 11 | 30 | 3.74 | 0.527 | 0.21 |
| 30 | 1 | 4278_0.5 | 0.48 | 45 | 214 | 4.5 | 0.332 | 0.119 |
| 30 | 1 | 4384_0.48 | 0.44 | 10 | 37 | 4.9 | 0.642 | 0.156 |
| 30 | 1 | 4395_0.48 | 0.42 | 15 | 44 | 6.75 | 0.451 | 0.376 |
| 30 | 1 | 4432_0.47 | 0.43 | 22 | 59 | 5.28 | 0.248 | 0.238 |
| 30 | 1 | 4467_0.46 | 0.45 | 16 | 36 | 5.44 | 0.356 | 0.154 |
| 30 | 1 | 4563_0.44 | 0.42 | 12 | 38 | 5.22 | 0.467 | 0.103 |
| 30 | 1 | 4618_0.43 | 0.38 | 16 | 75 | 10.08 | 0.638 | 0.227 |
| 30 | 1 | 4653_0.42 | 0.37 | 18 | 36 | 4.14 | 0.228 | 0.144 |
| 30 | 1 | 4663_0.42 | 0.32 | 16 | 53 | 5.92 | 0.369 | 0.142 |
| 30 | 1 | 4679_0.41 | 0.39 | 12 | 55 | 7.8 | 0.656 | 0.247 |
| 30 | 1 | 4708_0.41 | 0.24 | 18 | 62 | 6.84 | 0.378 | 0.301 |
| 30 | 1 | 4714_0.4 | 0.39 | 11 | 32 | 5.115 | 0.506 | 0.475 |
| 30 | 1 | 4730_0.4 | 0.34 | 16 | 44 | 5.92 | 0.369 | 0.322 |
| 30 | 1 | 4750_0.39 | 0.38 | 18 | 43 | 3.6 | 0.209 | 0.179 |
| 30 | 1 | 4757_0.39 | 0.37 | 32 | 82 | 5.12 | 0.168 | 0.0951 |
| 30 | 1 | 4860_0.36 | 0.35 | 27 | 95 | 6.21 | 0.238 | 0.128 |
| 30 | 1 | 4872_0.36 | 0.31 | 11 | 42 | 3.85 | 0.363 | 0.147 |
| 30 | 1 | 4998_0.31 | 0.29 | 12 | 38 | 4.62 | 0.397 | 0.396 |
| 30 | 2 | 2043_0.92 | 0.91 | 124 | 3950 | 8.68 | 0.921 | 0.0534 |
| 30 | 2 | 2318_0.89 | 0.88 | 222 | 10937 | 55.5 | 0.87 | 0.0705 |
| 30 | 2 | 2398_0.88 | 0.87 | 268 | 17510 | 91.12 | 0.871 | 0.0748 |
| 30 | 2 | 2406_0.88 | 0.86 | 31 | 194 | 8.37 | 0.85 | 0.14 |
| 30 | 2 | 2640_0.85 | 0.6 | 23 | 232 | 20.47 | 0.97 | 0.0148 |
| 30 | 2 | 2667_0.84 | 0.83 | 246 | 10952 | 9.84 | 0.772 | 0.117 |
| 30 | 2 | 2849_0.81 | 0.6 | 12 | 34 | 7.56 | 0.807 | 0.074 |
| 30 | 2 | 2943_0.79 | 0.52 | 18 | 107 | 12.78 | 0.921 | 0.06 |
| 30 | 2 | 3228_0.73 | 0.66 | 16 | 58 | 7.44 | 0.696 | 0.178 |
| 30 | 2 | 3243_0.73 | 0.54 | 13 | 39 | 9.035 | 0.702 | 0.105 |
| 30 | 2 | 3334_0.71 | 0.64 | 30 | 146 | 6.9 | 0.611 | 0.151 |
| 30 | 2 | 3415_0.69 | 0.68 | 57 | 273 | 10.26 | 0.49 | 0.117 |
| 30 | 2 | 3469_0.68 | 0.64 | 32 | 202 | 8.32 | 0.623 | 0.266 |
| 30 | 2 | 3508_0.67 | 0.65 | 62 | 402 | 17.36 | 0.442 | 0.161 |
| 30 | 2 | 3596_0.65 | 0.62 | 22 | 106 | 11.44 | 0.62 | 0.15 |
| 30 | 2 | 3608_0.65 | 0.48 | 11 | 32 | 8.36 | 0.768 | 0.182 |
| 30 | 2 | 3653_0.64 | 0.49 | 10 | 37 | 9.7 | 0.98 | 0.0129 |
| 30 | 2 | 3671_0.63 | 0.62 | 36 | 124 | 10.08 | 0.423 | 0.141 |
| 30 | 2 | 3693_0.63 | 0.51 | 13 | 39 | 7.605 | 0.618 | 0.162 |
| 30 | 2 | 3736_0.62 | 0.48 | 10 | 37 | 6.7 | 0.936 | 0.0508 |
| 30 | 2 | 3767_0.61 | 0.59 | 17 | 53 | 6.46 | 0.527 | 0.226 |
| 30 | 2 | 3852_0.59 | 0.58 | 26 | 96 | 10.92 | 0.445 | 0.179 |
| 30 | 2 | 3888_0.59 | 0.37 | 18 | 81 | 11.88 | 0.674 | 0.112 |
| 30 | 2 | 3942_0.57 | 0.56 | 24 | 91 | 11.4 | 0.487 | 0.189 |
| 30 | 2 | 3944_0.57 | 0.56 | 19 | 55 | 6.46 | 0.38 | 0.17 |
| 30 | 2 | 3995_0.56 | 0.54 | 25 | 131 | 6.25 | 0.473 | 0.219 |
| 30 | 2 | 4050_0.55 | 0.49 | 17 | 36 | 5.27 | 0.362 | 0.0957 |
| 30 | 2 | 4074_0.55 | 0.29 | 11 | 44 | 7.59 | 0.702 | 0.153 |
| 30 | 2 | 4127_0.54 | 0.29 | 15 | 63 | 9 | 0.614 | 0.168 |
| 30 | 2 | 4241_0.51 | 0.47 | 14 | 57 | 9.1 | 0.657 | 0.203 |
| 30 | 2 | 4257_0.51 | 0.42 | 17 | 97 | 11.05 | 0.649 | 0.299 |
| 30 | 2 | 4266_0.51 | 0.33 | 10 | 30 | 7.3 | 0.732 | 0.245 |
| 30 | 2 | 4280_0.5 | 0.48 | 30 | 88 | 8.1 | 0.306 | 0.144 |
| 30 | 2 | 4340_0.49 | 0.42 | 21 | 95 | 8.61 | 0.43 | 0.415 |
| 30 | 2 | 4395_0.48 | 0.42 | 15 | 44 | 6.75 | 0.451 | 0.376 |
| 30 | 2 | 4432_0.47 | 0.43 | 22 | 59 | 5.28 | 0.248 | 0.238 |
| 30 | 2 | 4463_0.46 | 0.45 | 71 | 334 | 13.49 | 0.192 | 0.124 |
| 30 | 2 | 4467_0.46 | 0.45 | 16 | 36 | 5.44 | 0.356 | 0.154 |
| 30 | 2 | 4470_0.46 | 0.44 | 61 | 333 | 14.64 | 0.247 | 0.108 |
| 30 | 2 | 4520_0.45 | 0.43 | 61 | 186 | 6.1 | 0.114 | 0.113 |
| 30 | 2 | 4618_0.43 | 0.38 | 16 | 75 | 10.08 | 0.638 | 0.227 |
| 30 | 2 | 4663_0.42 | 0.32 | 16 | 53 | 5.92 | 0.369 | 0.142 |
| 30 | 2 | 4679_0.41 | 0.39 | 12 | 55 | 7.8 | 0.656 | 0.247 |
| 30 | 2 | 4708_0.41 | 0.24 | 18 | 62 | 6.84 | 0.378 | 0.301 |
| 30 | 2 | 4730_0.4 | 0.34 | 16 | 44 | 5.92 | 0.369 | 0.322 |
| 30 | 2 | 4860_0.36 | 0.35 | 27 | 95 | 6.21 | 0.238 | 0.128 |
| 30 | 5 | 2317_0.89 | 0.88 | 199 | 5105 | 39.8 | 0.811 | 0.0719 |
| 30 | 5 | 2318_0.89 | 0.88 | 222 | 10937 | 55.5 | 0.87 | 0.0705 |
| 30 | 5 | 2398_0.88 | 0.87 | 268 | 17510 | 91.12 | 0.871 | 0.0748 |
| 30 | 5 | 2640_0.85 | 0.6 | 23 | 232 | 20.47 | 0.97 | 0.0148 |
| 30 | 5 | 2943_0.79 | 0.52 | 18 | 107 | 12.78 | 0.921 | 0.06 |
| 30 | 5 | 3414_0.69 | 0.68 | 347 | 21468 | 34.7 | 0.579 | 0.177 |
| 30 | 5 | 3508_0.67 | 0.65 | 62 | 402 | 17.36 | 0.442 | 0.161 |
| 30 | 5 | 3596_0.65 | 0.62 | 22 | 106 | 11.44 | 0.62 | 0.15 |
| 30 | 5 | 3852_0.59 | 0.58 | 26 | 96 | 10.92 | 0.445 | 0.179 |
| 30 | 5 | 3888_0.59 | 0.37 | 18 | 81 | 11.88 | 0.674 | 0.112 |
| 30 | 5 | 3942_0.57 | 0.56 | 24 | 91 | 11.4 | 0.487 | 0.189 |
| 30 | 5 | 4257_0.51 | 0.42 | 17 | 97 | 11.05 | 0.649 | 0.299 |
| 30 | 5 | 4463_0.46 | 0.45 | 71 | 334 | 13.49 | 0.192 | 0.124 |
| 30 | 5 | 4470_0.46 | 0.44 | 61 | 333 | 14.64 | 0.247 | 0.108 |
| 30 | 5 | 4583_0.44 | 0.35 | 72 | 973 | 23.76 | 0.337 | 0.173 |
| 30 | 10 | 2317_0.89 | 0.88 | 199 | 5105 | 39.8 | 0.811 | 0.0719 |
| 30 | 10 | 2318_0.89 | 0.88 | 222 | 10937 | 55.5 | 0.87 | 0.0705 |
| 30 | 10 | 2398_0.88 | 0.87 | 268 | 17510 | 91.12 | 0.871 | 0.0748 |
| 30 | 10 | 2640_0.85 | 0.6 | 23 | 232 | 20.47 | 0.97 | 0.0148 |
| 30 | 10 | 3414_0.69 | 0.68 | 347 | 21468 | 34.7 | 0.579 | 0.177 |
| 30 | 10 | 4583_0.44 | 0.35 | 72 | 973 | 23.76 | 0.337 | 0.173 |
| 60 | 5 | 2317_0.89 | 0.88 | 199 | 5105 | 39.8 | 0.811 | 0.0719 |
| 60 | 5 | 2318_0.89 | 0.88 | 222 | 10937 | 55.5 | 0.87 | 0.0705 |
| 60 | 5 | 2398_0.88 | 0.87 | 268 | 17510 | 91.12 | 0.871 | 0.0748 |
| 60 | 5 | 2640_0.85 | 0.6 | 23 | 232 | 20.47 | 0.97 | 0.0148 |
| 60 | 5 | 2943_0.79 | 0.52 | 18 | 107 | 12.78 | 0.921 | 0.06 |
| 60 | 5 | 3414_0.69 | 0.68 | 347 | 21468 | 34.7 | 0.579 | 0.177 |
| 60 | 5 | 3508_0.67 | 0.65 | 62 | 402 | 17.36 | 0.442 | 0.161 |
| 60 | 5 | 3596_0.65 | 0.62 | 22 | 106 | 11.44 | 0.62 | 0.15 |
| 60 | 5 | 3888_0.59 | 0.37 | 18 | 81 | 11.88 | 0.674 | 0.112 |
| 60 | 5 | 3942_0.57 | 0.56 | 24 | 91 | 11.4 | 0.487 | 0.189 |
| 60 | 5 | 4257_0.51 | 0.42 | 17 | 97 | 11.05 | 0.649 | 0.299 |
| 60 | 5 | 4463_0.46 | 0.45 | 71 | 334 | 13.49 | 0.192 | 0.124 |
| 60 | 5 | 4470_0.46 | 0.44 | 61 | 333 | 14.64 | 0.247 | 0.108 |
| 60 | 5 | 4583_0.44 | 0.35 | 72 | 973 | 23.76 | 0.337 | 0.173 |
| 100 | 10 | 2317_0.89 | 0.88 | 199 | 5105 | 39.8 | 0.811 | 0.0719 |
| 100 | 10 | 2318_0.89 | 0.88 | 222 | 10937 | 55.5 | 0.87 | 0.0705 |
| 100 | 10 | 2398_0.88 | 0.87 | 268 | 17510 | 91.12 | 0.871 | 0.0748 |
| 100 | 10 | 3414_0.69 | 0.68 | 347 | 21468 | 34.7 | 0.579 | 0.177 |
| 100 | 10 | 4583_0.44 | 0.35 | 72 | 973 | 23.76 | 0.337 | 0.173 |
| 200 | 1 | 2043_0.92 | 0.91 | 124 | 3950 | 8.68 | 0.921 | 0.0534 |
| 200 | 1 | 2318_0.89 | 0.88 | 222 | 10937 | 55.5 | 0.87 | 0.0705 |
| 200 | 1 | 2398_0.88 | 0.87 | 268 | 17510 | 91.12 | 0.871 | 0.0748 |
| 200 | 1 | 2401_0.88 | 0.87 | 221 | 7954 | 4.42 | 0.816 | 0.095 |
| 200 | 1 | 2640_0.85 | 0.6 | 23 | 232 | 20.47 | 0.97 | 0.0148 |
| 200 | 1 | 3415_0.69 | 0.68 | 57 | 273 | 10.26 | 0.49 | 0.117 |
| 200 | 1 | 3469_0.68 | 0.64 | 32 | 202 | 8.32 | 0.623 | 0.266 |
| 200 | 1 | 3508_0.67 | 0.65 | 62 | 402 | 17.36 | 0.442 | 0.161 |
| 200 | 1 | 4239_0.51 | 0.49 | 40 | 218 | 5.8 | 0.235 | 0.227 |
| 200 | 1 | 4240_0.51 | 0.48 | 59 | 273 | 8.26 | 0.165 | 0.165 |
| 200 | 1 | 4278_0.5 | 0.48 | 45 | 214 | 4.5 | 0.332 | 0.119 |
| 200 | 1 | 4470_0.46 | 0.44 | 61 | 333 | 14.64 | 0.247 | 0.108 |
| 200 | 2 | 2043_0.92 | 0.91 | 124 | 3950 | 8.68 | 0.921 | 0.0534 |
| 200 | 2 | 2318_0.89 | 0.88 | 222 | 10937 | 55.5 | 0.87 | 0.0705 |
| 200 | 2 | 2398_0.88 | 0.87 | 268 | 17510 | 91.12 | 0.871 | 0.0748 |
| 200 | 2 | 2640_0.85 | 0.6 | 23 | 232 | 20.47 | 0.97 | 0.0148 |
| 200 | 2 | 2667_0.84 | 0.83 | 246 | 10952 | 9.84 | 0.772 | 0.117 |
| 200 | 2 | 3415_0.69 | 0.68 | 57 | 273 | 10.26 | 0.49 | 0.117 |
| 200 | 2 | 3469_0.68 | 0.64 | 32 | 202 | 8.32 | 0.623 | 0.266 |
| 200 | 2 | 3508_0.67 | 0.65 | 62 | 402 | 17.36 | 0.442 | 0.161 |
| 200 | 2 | 4240_0.51 | 0.48 | 59 | 273 | 8.26 | 0.165 | 0.165 |
| 200 | 2 | 4463_0.46 | 0.45 | 71 | 334 | 13.49 | 0.192 | 0.124 |
| 200 | 2 | 4470_0.46 | 0.44 | 61 | 333 | 14.64 | 0.247 | 0.108 |
| 200 | 3 | 2043_0.92 | 0.91 | 124 | 3950 | 8.68 | 0.921 | 0.0534 |
| 200 | 3 | 2318_0.89 | 0.88 | 222 | 10937 | 55.5 | 0.87 | 0.0705 |
| 200 | 3 | 2398_0.88 | 0.87 | 268 | 17510 | 91.12 | 0.871 | 0.0748 |
| 200 | 3 | 2640_0.85 | 0.6 | 23 | 232 | 20.47 | 0.97 | 0.0148 |
| 200 | 3 | 2667_0.84 | 0.83 | 246 | 10952 | 9.84 | 0.772 | 0.117 |
| 200 | 3 | 3415_0.69 | 0.68 | 57 | 273 | 10.26 | 0.49 | 0.117 |
| 200 | 3 | 3508_0.67 | 0.65 | 62 | 402 | 17.36 | 0.442 | 0.161 |
| 200 | 3 | 4463_0.46 | 0.45 | 71 | 334 | 13.49 | 0.192 | 0.124 |
| 200 | 3 | 4470_0.46 | 0.44 | 61 | 333 | 14.64 | 0.247 | 0.108 |
| 200 | 3 | 4583_0.44 | 0.35 | 72 | 973 | 23.76 | 0.337 | 0.173 |
| 200 | 4 | 2317_0.89 | 0.88 | 199 | 5105 | 39.8 | 0.811 | 0.0719 |
| 200 | 4 | 2318_0.89 | 0.88 | 222 | 10937 | 55.5 | 0.87 | 0.0705 |
| 200 | 4 | 2398_0.88 | 0.87 | 268 | 17510 | 91.12 | 0.871 | 0.0748 |
| 200 | 4 | 2640_0.85 | 0.6 | 23 | 232 | 20.47 | 0.97 | 0.0148 |
| 200 | 4 | 3414_0.69 | 0.68 | 347 | 21468 | 34.7 | 0.579 | 0.177 |
| 200 | 4 | 3508_0.67 | 0.65 | 62 | 402 | 17.36 | 0.442 | 0.161 |
| 200 | 4 | 4463_0.46 | 0.45 | 71 | 334 | 13.49 | 0.192 | 0.124 |
| 200 | 4 | 4470_0.46 | 0.44 | 61 | 333 | 14.64 | 0.247 | 0.108 |
| 200 | 4 | 4583_0.44 | 0.35 | 72 | 973 | 23.76 | 0.337 | 0.173 |
| 200 | 5 | 2317_0.89 | 0.88 | 199 | 5105 | 39.8 | 0.811 | 0.0719 |
| 200 | 5 | 2318_0.89 | 0.88 | 222 | 10937 | 55.5 | 0.87 | 0.0705 |
| 200 | 5 | 2398_0.88 | 0.87 | 268 | 17510 | 91.12 | 0.871 | 0.0748 |
| 200 | 5 | 2640_0.85 | 0.6 | 23 | 232 | 20.47 | 0.97 | 0.0148 |
| 200 | 5 | 3414_0.69 | 0.68 | 347 | 21468 | 34.7 | 0.579 | 0.177 |
| 200 | 5 | 3508_0.67 | 0.65 | 62 | 402 | 17.36 | 0.442 | 0.161 |
| 200 | 5 | 4463_0.46 | 0.45 | 71 | 334 | 13.49 | 0.192 | 0.124 |
| 200 | 5 | 4470_0.46 | 0.44 | 61 | 333 | 14.64 | 0.247 | 0.108 |
| 200 | 5 | 4583_0.44 | 0.35 | 72 | 973 | 23.76 | 0.337 | 0.173 |
| 200 | 6 | 2317_0.89 | 0.88 | 199 | 5105 | 39.8 | 0.811 | 0.0719 |
| 200 | 6 | 2318_0.89 | 0.88 | 222 | 10937 | 55.5 | 0.87 | 0.0705 |
| 200 | 6 | 2398_0.88 | 0.87 | 268 | 17510 | 91.12 | 0.871 | 0.0748 |
| 200 | 6 | 2640_0.85 | 0.6 | 23 | 232 | 20.47 | 0.97 | 0.0148 |
| 200 | 6 | 3414_0.69 | 0.68 | 347 | 21468 | 34.7 | 0.579 | 0.177 |
| 200 | 6 | 3508_0.67 | 0.65 | 62 | 402 | 17.36 | 0.442 | 0.161 |
| 200 | 6 | 4583_0.44 | 0.35 | 72 | 973 | 23.76 | 0.337 | 0.173 |
| 200 | 7 | 2317_0.89 | 0.88 | 199 | 5105 | 39.8 | 0.811 | 0.0719 |
| 200 | 7 | 2318_0.89 | 0.88 | 222 | 10937 | 55.5 | 0.87 | 0.0705 |
| 200 | 7 | 2398_0.88 | 0.87 | 268 | 17510 | 91.12 | 0.871 | 0.0748 |
| 200 | 7 | 2640_0.85 | 0.6 | 23 | 232 | 20.47 | 0.97 | 0.0148 |
| 200 | 7 | 3414_0.69 | 0.68 | 347 | 21468 | 34.7 | 0.579 | 0.177 |
| 200 | 7 | 3508_0.67 | 0.65 | 62 | 402 | 17.36 | 0.442 | 0.161 |
| 200 | 7 | 4583_0.44 | 0.35 | 72 | 973 | 23.76 | 0.337 | 0.173 |
| 200 | 8 | 2317_0.89 | 0.88 | 199 | 5105 | 39.8 | 0.811 | 0.0719 |
| 200 | 8 | 2318_0.89 | 0.88 | 222 | 10937 | 55.5 | 0.87 | 0.0705 |
| 200 | 8 | 2398_0.88 | 0.87 | 268 | 17510 | 91.12 | 0.871 | 0.0748 |
| 200 | 8 | 2640_0.85 | 0.6 | 23 | 232 | 20.47 | 0.97 | 0.0148 |
| 200 | 8 | 3414_0.69 | 0.68 | 347 | 21468 | 34.7 | 0.579 | 0.177 |
| 200 | 8 | 4583_0.44 | 0.35 | 72 | 973 | 23.76 | 0.337 | 0.173 |
| 200 | 10 | 2317_0.89 | 0.88 | 199 | 5105 | 39.8 | 0.811 | 0.0719 |
| 200 | 10 | 2318_0.89 | 0.88 | 222 | 10937 | 55.5 | 0.87 | 0.0705 |
| 200 | 10 | 2398_0.88 | 0.87 | 268 | 17510 | 91.12 | 0.871 | 0.0748 |
| 200 | 10 | 3414_0.69 | 0.68 | 347 | 21468 | 34.7 | 0.579 | 0.177 |
| 400 | 1 | 2043_0.92 | 0.91 | 124 | 3950 | 8.68 | 0.921 | 0.0534 |
| 400 | 1 | 2318_0.89 | 0.88 | 222 | 10937 | 55.5 | 0.87 | 0.0705 |
| 400 | 1 | 2398_0.88 | 0.87 | 268 | 17510 | 91.12 | 0.871 | 0.0748 |
| 400 | 1 | 2667_0.84 | 0.83 | 246 | 10952 | 9.84 | 0.772 | 0.117 |
| 400 | 1 | 3508_0.67 | 0.65 | 62 | 402 | 17.36 | 0.442 | 0.161 |
| 400 | 1 | 3658_0.64 | 0.44 | 62 | 429 | 6.2 | 0.43 | 0.162 |
| 400 | 2 | 2043_0.92 | 0.91 | 124 | 3950 | 8.68 | 0.921 | 0.0534 |
| 400 | 2 | 2318_0.89 | 0.88 | 222 | 10937 | 55.5 | 0.87 | 0.0705 |
| 400 | 2 | 2398_0.88 | 0.87 | 268 | 17510 | 91.12 | 0.871 | 0.0748 |
| 400 | 2 | 2667_0.84 | 0.83 | 246 | 10952 | 9.84 | 0.772 | 0.117 |
| 400 | 2 | 3508_0.67 | 0.65 | 62 | 402 | 17.36 | 0.442 | 0.161 |
| 400 | 2 | 4583_0.44 | 0.35 | 72 | 973 | 23.76 | 0.337 | 0.173 |
| 400 | 3 | 2317_0.89 | 0.88 | 199 | 5105 | 39.8 | 0.811 | 0.0719 |
| 400 | 3 | 2318_0.89 | 0.88 | 222 | 10937 | 55.5 | 0.87 | 0.0705 |
| 400 | 3 | 2398_0.88 | 0.87 | 268 | 17510 | 91.12 | 0.871 | 0.0748 |
| 400 | 3 | 2667_0.84 | 0.83 | 246 | 10952 | 9.84 | 0.772 | 0.117 |
| 400 | 3 | 3508_0.67 | 0.65 | 62 | 402 | 17.36 | 0.442 | 0.161 |
| 400 | 3 | 4583_0.44 | 0.35 | 72 | 973 | 23.76 | 0.337 | 0.173 |
| 400 | 4 | 2317_0.89 | 0.88 | 199 | 5105 | 39.8 | 0.811 | 0.0719 |
| 400 | 4 | 2318_0.89 | 0.88 | 222 | 10937 | 55.5 | 0.87 | 0.0705 |
| 400 | 4 | 2398_0.88 | 0.87 | 268 | 17510 | 91.12 | 0.871 | 0.0748 |
| 400 | 4 | 3414_0.69 | 0.68 | 347 | 21468 | 34.7 | 0.579 | 0.177 |
| 400 | 4 | 3508_0.67 | 0.65 | 62 | 402 | 17.36 | 0.442 | 0.161 |
| 400 | 4 | 4583_0.44 | 0.35 | 72 | 973 | 23.76 | 0.337 | 0.173 |
| 400 | 5 | 2317_0.89 | 0.88 | 199 | 5105 | 39.8 | 0.811 | 0.0719 |
| 400 | 5 | 2318_0.89 | 0.88 | 222 | 10937 | 55.5 | 0.87 | 0.0705 |
| 400 | 5 | 2398_0.88 | 0.87 | 268 | 17510 | 91.12 | 0.871 | 0.0748 |
| 400 | 5 | 3414_0.69 | 0.68 | 347 | 21468 | 34.7 | 0.579 | 0.177 |
| 400 | 5 | 3508_0.67 | 0.65 | 62 | 402 | 17.36 | 0.442 | 0.161 |
| 400 | 5 | 4583_0.44 | 0.35 | 72 | 973 | 23.76 | 0.337 | 0.173 |
| 400 | 6 | 2317_0.89 | 0.88 | 199 | 5105 | 39.8 | 0.811 | 0.0719 |
| 400 | 6 | 2318_0.89 | 0.88 | 222 | 10937 | 55.5 | 0.87 | 0.0705 |
| 400 | 6 | 2398_0.88 | 0.87 | 268 | 17510 | 91.12 | 0.871 | 0.0748 |
| 400 | 6 | 3414_0.69 | 0.68 | 347 | 21468 | 34.7 | 0.579 | 0.177 |
| 400 | 6 | 3508_0.67 | 0.65 | 62 | 402 | 17.36 | 0.442 | 0.161 |
| 400 | 6 | 4583_0.44 | 0.35 | 72 | 973 | 23.76 | 0.337 | 0.173 |
| 400 | 7 | 2317_0.89 | 0.88 | 199 | 5105 | 39.8 | 0.811 | 0.0719 |
| 400 | 7 | 2318_0.89 | 0.88 | 222 | 10937 | 55.5 | 0.87 | 0.0705 |
| 400 | 7 | 2398_0.88 | 0.87 | 268 | 17510 | 91.12 | 0.871 | 0.0748 |
| 400 | 7 | 3414_0.69 | 0.68 | 347 | 21468 | 34.7 | 0.579 | 0.177 |
| 400 | 7 | 4583_0.44 | 0.35 | 72 | 973 | 23.76 | 0.337 | 0.173 |
| 400 | 8 | 2317_0.89 | 0.88 | 199 | 5105 | 39.8 | 0.811 | 0.0719 |
| 400 | 8 | 2318_0.89 | 0.88 | 222 | 10937 | 55.5 | 0.87 | 0.0705 |
| 400 | 8 | 2398_0.88 | 0.87 | 268 | 17510 | 91.12 | 0.871 | 0.0748 |
| 400 | 8 | 3414_0.69 | 0.68 | 347 | 21468 | 34.7 | 0.579 | 0.177 |
| 400 | 8 | 4583_0.44 | 0.35 | 72 | 973 | 23.76 | 0.337 | 0.173 |
| 600 | 1 | 2043_0.92 | 0.91 | 124 | 3950 | 8.68 | 0.921 | 0.0534 |
| 600 | 1 | 2318_0.89 | 0.88 | 222 | 10937 | 55.5 | 0.87 | 0.0705 |
| 600 | 1 | 2398_0.88 | 0.87 | 268 | 17510 | 91.12 | 0.871 | 0.0748 |
| 600 | 1 | 2667_0.84 | 0.83 | 246 | 10952 | 9.84 | 0.772 | 0.117 |
| 600 | 1 | 4583_0.44 | 0.35 | 72 | 973 | 23.76 | 0.337 | 0.173 |
| 600 | 2 | 2043_0.92 | 0.91 | 124 | 3950 | 8.68 | 0.921 | 0.0534 |
| 600 | 2 | 2318_0.89 | 0.88 | 222 | 10937 | 55.5 | 0.87 | 0.0705 |
| 600 | 2 | 2398_0.88 | 0.87 | 268 | 17510 | 91.12 | 0.871 | 0.0748 |
| 600 | 2 | 2667_0.84 | 0.83 | 246 | 10952 | 9.84 | 0.772 | 0.117 |
| 600 | 2 | 4583_0.44 | 0.35 | 72 | 973 | 23.76 | 0.337 | 0.173 |
| 600 | 3 | 2317_0.89 | 0.88 | 199 | 5105 | 39.8 | 0.811 | 0.0719 |
| 600 | 3 | 2318_0.89 | 0.88 | 222 | 10937 | 55.5 | 0.87 | 0.0705 |
| 600 | 3 | 2398_0.88 | 0.87 | 268 | 17510 | 91.12 | 0.871 | 0.0748 |
| 600 | 3 | 2667_0.84 | 0.83 | 246 | 10952 | 9.84 | 0.772 | 0.117 |
| 600 | 3 | 4583_0.44 | 0.35 | 72 | 973 | 23.76 | 0.337 | 0.173 |
| 600 | 4 | 2317_0.89 | 0.88 | 199 | 5105 | 39.8 | 0.811 | 0.0719 |
| 600 | 4 | 2318_0.89 | 0.88 | 222 | 10937 | 55.5 | 0.87 | 0.0705 |
| 600 | 4 | 2398_0.88 | 0.87 | 268 | 17510 | 91.12 | 0.871 | 0.0748 |
| 600 | 4 | 3414_0.69 | 0.68 | 347 | 21468 | 34.7 | 0.579 | 0.177 |
| 600 | 4 | 4583_0.44 | 0.35 | 72 | 973 | 23.76 | 0.337 | 0.173 |
| 600 | 5 | 2317_0.89 | 0.88 | 199 | 5105 | 39.8 | 0.811 | 0.0719 |
| 600 | 5 | 2318_0.89 | 0.88 | 222 | 10937 | 55.5 | 0.87 | 0.0705 |
| 600 | 5 | 2398_0.88 | 0.87 | 268 | 17510 | 91.12 | 0.871 | 0.0748 |
| 600 | 5 | 3414_0.69 | 0.68 | 347 | 21468 | 34.7 | 0.579 | 0.177 |
| 600 | 5 | 4583_0.44 | 0.35 | 72 | 973 | 23.76 | 0.337 | 0.173 |
| 600 | 6 | 2317_0.89 | 0.88 | 199 | 5105 | 39.8 | 0.811 | 0.0719 |
| 600 | 6 | 2318_0.89 | 0.88 | 222 | 10937 | 55.5 | 0.87 | 0.0705 |
| 600 | 6 | 2398_0.88 | 0.87 | 268 | 17510 | 91.12 | 0.871 | 0.0748 |
| 600 | 6 | 3414_0.69 | 0.68 | 347 | 21468 | 34.7 | 0.579 | 0.177 |
| 600 | 6 | 4583_0.44 | 0.35 | 72 | 973 | 23.76 | 0.337 | 0.173 |
| 600 | 7 | 2317_0.89 | 0.88 | 199 | 5105 | 39.8 | 0.811 | 0.0719 |
| 600 | 7 | 2318_0.89 | 0.88 | 222 | 10937 | 55.5 | 0.87 | 0.0705 |
| 600 | 7 | 2398_0.88 | 0.87 | 268 | 17510 | 91.12 | 0.871 | 0.0748 |
| 600 | 7 | 3414_0.69 | 0.68 | 347 | 21468 | 34.7 | 0.579 | 0.177 |
| 600 | 7 | 4583_0.44 | 0.35 | 72 | 973 | 23.76 | 0.337 | 0.173 |
| 600 | 8 | 2317_0.89 | 0.88 | 199 | 5105 | 39.8 | 0.811 | 0.0719 |
| 600 | 8 | 2318_0.89 | 0.88 | 222 | 10937 | 55.5 | 0.87 | 0.0705 |
| 600 | 8 | 2398_0.88 | 0.87 | 268 | 17510 | 91.12 | 0.871 | 0.0748 |
| 600 | 8 | 3414_0.69 | 0.68 | 347 | 21468 | 34.7 | 0.579 | 0.177 |
| 600 | 8 | 4583_0.44 | 0.35 | 72 | 973 | 23.76 | 0.337 | 0.173 |
| 1000 | 10 | 2317_0.89 | 0.88 | 199 | 5105 | 39.8 | 0.811 | 0.0719 |
| 1000 | 10 | 2318_0.89 | 0.88 | 222 | 10937 | 55.5 | 0.87 | 0.0705 |
| 1000 | 10 | 2398_0.88 | 0.87 | 268 | 17510 | 91.12 | 0.871 | 0.0748 |
| 1000 | 10 | 3414_0.69 | 0.68 | 347 | 21468 | 34.7 | 0.579 | 0.177 |
| 4000 | 4 | 2317_0.89 | 0.88 | 199 | 5105 | 39.8 | 0.811 | 0.0719 |
| 4000 | 4 | 2318_0.89 | 0.88 | 222 | 10937 | 55.5 | 0.87 | 0.0705 |
| 4000 | 4 | 2398_0.88 | 0.87 | 268 | 17510 | 91.12 | 0.871 | 0.0748 |
| 4000 | 4 | 3414_0.69 | 0.68 | 347 | 21468 | 34.7 | 0.579 | 0.177 |

**Supporting Table 2.** Cline parameter estimates and confidence intervals (C.I.s) for the best cline model of R arrangement frequency along the transect for each of the four inversions for males and females. Crab frequency and Wave frequency indicate the fitted frequencies of the R arrangement in the Crab and the Wave ends of the transect. Best cline model was taken as the model with the lowest AIC value (see Supporting Table 3).

| Inversion | Sex | Best model | Crab frequency | | | Wave frequency | | | Centre (m) | | | Width (m) | | |
| --- | --- | --- | --- | --- | --- | --- | --- | --- | --- | --- | --- | --- | --- | --- |
|  |  |  | Estimate | Lower C.I. | Upper C.I. | Estimate | Lower C.I. | Upper C.I. | Estimate | Lower C.I. | Upper C.I. | Estimate | Lower C.I. | Upper C.I. |
| 12.1 | Female | Full | 0.996 | 0.981 | - | 0.720 | 0.630 | 0.798 | 87.589 | 82.853 | 95.606 | 6.08 | 0.729 | 34.865 |
|  | Male | Full | 1.000 | 1.00 | - | 0.750 | 0.674 | 0.817 | 85.118 | 82.031 | 85.666 | 0.104 | 0.00 | - |
| 12.2 | Female | No cline | 0.510 | 0.461 | 0.558 | 0.510 | 0.461 | 0.558 | N/A | N/A | N/A | N/A | N/A | N/A |
|  | Male | Full | 0.983 | - | - | 0.663 | - | - | 83.761 | - | - | 4.286 | - | - |
| 12.3 | Female | Wave-constrained | 0.503 | 0.434 | 0.597 | 0.042 | 0.024 | 0.066 | 90.329 | 83.648 | 96.270 | 23.000 | 0.476 | 54.298 |
|  | Male | Wave-constrained | 0.042 | 0.024 | 0.066 | 0.042 | 0.024 | 0.066 | 90.329 | 83.648 | 96.270 | 23.000 | 0.476 | 54.298 |
| 12.4 | Female | Wave-constrained | 0.603 | 0.517 | 0.666 | 0.081 | 0.043 | 0.132 | 90.433 | 87.613 | 93.917 | 10.122 | 0.758 | 21.155 |
|  | Male | Wave-constrained | 0.326 | 0.268 | 0.389 | 0.081 | 0.043 | 0.132 | 90.433 | 87.613 | 93.917 | 10.122 | 0.758 | 21.155 |

**Supporting Table 3.** AIC values for the full cline models and the four alternative models for each inversion. For models fitted separately to males and females (‘full model’ and ‘no cline’), the sum of male and female AIC values for each combination of these is included. The model (or combination of models) with the lowest AIC value for males and females for each inversion is highlighted in bold.

| Inversion | Male + female ‘full model’ | Male + female ‘no cline’ | Male ‘full model’ + female ‘no cline’ | Female ‘full model’ + male ‘no cline’ | ‘Combined’ cline | ‘Constrained’ cline | ‘Wave-constrained’ cline |
| --- | --- | --- | --- | --- | --- | --- | --- |
| 12.1 | **284.61** | 406.58 | 347.49 | 345.70 | 285.04 | 288.08 | N/A |
| 12.2 | 516.26 | 570.70 | **510.57** | 576.42 | 667.80 | 512.37 | 516.72 |
| 12.3 | 381.30 | 440.40 | 438.65 | 383.05 | 522.35 | 384.50 | **381.05** |
| 12.4 | 599.04 | 694.59 | 674.06 | 619.57 | 634.06 | 597.57 | **596.31** |

**Supporting Table 4.** The number of individuals of each sex and ecotype of each inversion genotype for the four inversions. ‘-‘ indicates zero snails.

| Inversion | Sex | RR | | RA | | AA | |
| --- | --- | --- | --- | --- | --- | --- | --- |
|  |  | Crab | Wave | Crab | Wave | Crab | Wave |
| 12.1 | Female | 90 | 31 | - | 22 | - | 5 |
|  | Male | 62 | 23 | - | 18 | - | 1 |
| 12.2 | Female | 1 | 12 | 88 | 32 | 1 | 14 |
|  | Male | 60 | 17 | 2 | 21 | - | 4 |
| 12.3 | Female | 1 | 1 | 88 | 10 | 1 | 47 |
|  | Male | - | - | 2 | 3 | 60 | 39 |
| 12.4 | Female | 22 | 1 | 66 | 13 | 1 | 44 |
|  | Male | 7 | - | 26 | 6 | 29 | 36 |

**Supporting Table 5**. The best fitting models (any with ΔAIC<2 compared to the best model) for each of the four inversions, and weighting when models are averaged.

| Inversion | Ecotype | Genotype | Sex | Ecotype: genotype | Ecotype: sex | Genotype: sex | Ecotype: genotype:sex | df | AICc | ΔAIC | Weight |
| --- | --- | --- | --- | --- | --- | --- | --- | --- | --- | --- | --- |
| LGC12.1 |  | + |  |  |  |  |  | 82 | 2650.240 | 0 | 1 |
| LGC12.2 | + | + | + | + | + |  |  | 86 | 4975.964 | 0 | 1 |
| LGC12.3 |  | + | + |  |  | + |  | 41 | 3848.390 | 0 | 0.419 |
| LGC12.3 | + | + | + |  | + | + |  | 43 | 3849.118 | 0.728 | 0.291 |
| LGC12.3 | + | + | + |  |  | + |  | 42 | 3849.123 | 0.734 | 0.290 |
| LGC12.4 |  | + |  |  |  |  |  | 82 | 5467.944 | 0 | 0.320 |
| LGC12.4 | + | + |  |  |  |  |  | 83 | 5468.456 | 0.511 | 0.247 |
| LGC12.4 | + | + | + |  |  |  |  | 84 | 5469.345 | 1.401 | 0.159 |
| LGC12.4 |  | + | + |  |  |  |  | 83 | 5469.516 | 1.571 | 0.146 |
| LGC12.4 | + | + |  | + |  |  |  | 84 | 5469.764 | 1.819 | 0.129 |

**Supporting Table 6.** Model estimates for the best fitting model for each inversion. Model-weighted average estimates are presented for inversions with more than one best fitting model (see Supporting Table 5). Estimates and standard errors are for log-transformed data.

| Inversion | Coefficients | Estimate | Standard Error |
| --- | --- | --- | --- |
| LGC12.1 | Intercept | -3.9759 | 0.0702 |
| LGC12.1 | GenotypeAA | -0.8303 | 0.1197 |
| LGC12.1 | GenotypeRR | -0.2728 | 0.0823 |
| LGC12.2 | Intercept | -4.2778 | 0.0656 |
| LGC12.2 | EcotypeC | -0.2615 | 0.0681 |
| LGC12.2 | GenotypeAA | -0.4179 | 0.1024 |
| LGC12.2 | GenotypeRR | -0.2011 | 0.0543 |
| LGC12.2 | SexM | -0.0021 | 0.0450 |
| LGC12.2 | EcotypeC:genotypeRR | -0.2933 | 0.0977 |
| LGC12.2 | EcotypeC:sexM | 0.4088 | 0.0940 |
| LGC12.3 | Intercept | -4.4040 | 0.0718 |
| LGC12.3 | GenotypeAA | 0.0580 | 0.0805 |
| LGC12.3 | SexM | 0.2584 | 0.0776 |
| LGC12.3 | GenotypeAA:sexM | -0.2356 | 0.0957 |
| LGC12.3 | EcotypeC | -0.0742 | 0.0911 |
| LGC12.3 | EcotypeC:sexM | 0.0429 | 0.0852 |
| LGC12.4 | Intercept | -4.0724 | 0.0685 |
| LGC12.4 | GenotypeAA | -0.2680 | 0.0586 |
| LGC12.4 | GenotypeRR | -0.9312 | 0.1368 |
| LGC12.4 | EcotypeC | -0.0380 | 0.0477 |
| LGC12.4 | SexM | 0.0120 | 0.0272 |
| LGC12.4 | EcotypeC:genotypeAA | 0.0111 | 0.0403 |

**Supporting Figure 1**. The proportions of each sex that are heterozygous at SNPs on each of the 17 linkage groups in the two ecotypes. Hexagons with a greater density of SNPs are shaded lighter grey. SNPs with a greater difference in heterozygosity between the sexes are further from the 1:1 line (neutral expectation of equal heterozygote proportions between the sexes). The 1% of SNPs with the most negative residuals in Wave are marked by orange circles; the insert shows the distribution of these SNPs across the 17 linkage groups.

**Supporting Figure 2. A)** PC1 vs PC2 of a PCA of SNPs in the central region of LG12 covered by three LD clusters, for females and males. **B)** Plot showing the SNP genotypes along LG12 for every individual (each column shows an individual’s haplotype), in order along the sampling transect, for females and males. Red horizontal dashed lines denote the four different regions of LG12 (Figure 2)- the central region of LG12 used in **A)** corresponds to the two central sections in **B)**.

**Supporting Figure 3.** PC1 vs PC2 of a PCA of SNPs for all individuals (both sexes and ecotypes) for each putative inversion- LGC12.1 (0-32.8cM); LGC12.2 (33.0-43.8cM); LGC12.3 (43.8-48.7cM); and LGC12.4 (48.7-60.0cM). The three distinct clusters along PC1 that represent the three inversion genotypes are visible for all regions.

**Supporting Figure 4.** π **(A)** and d_XY_ **(B)** per contig in/between groups of individuals of each combination of inversion genotype, ecotype and sex for the four inversions. The first letter of each x axis label refers to the sex (F- female; M- male); the second part refers to the genotype (AA homozygote; RA heterozygote; RR homozygote); and the last letter refers to the ecotype (C- Crab; W- Wave) for each group. In **(B)**, labels include the two group codes for the two groups in each d_XY_ comparison.
