# Supplementary figures and images for: Differing associations between sex determination and sex‐linked inversions in two ecotypes of Littorina saxatilis
Source: Evol Lett. 2022 Aug 12;6(5):358–74. doi: 10.1002/evl3.295 (PMC9554762; doi:10.1002/evl3.295)

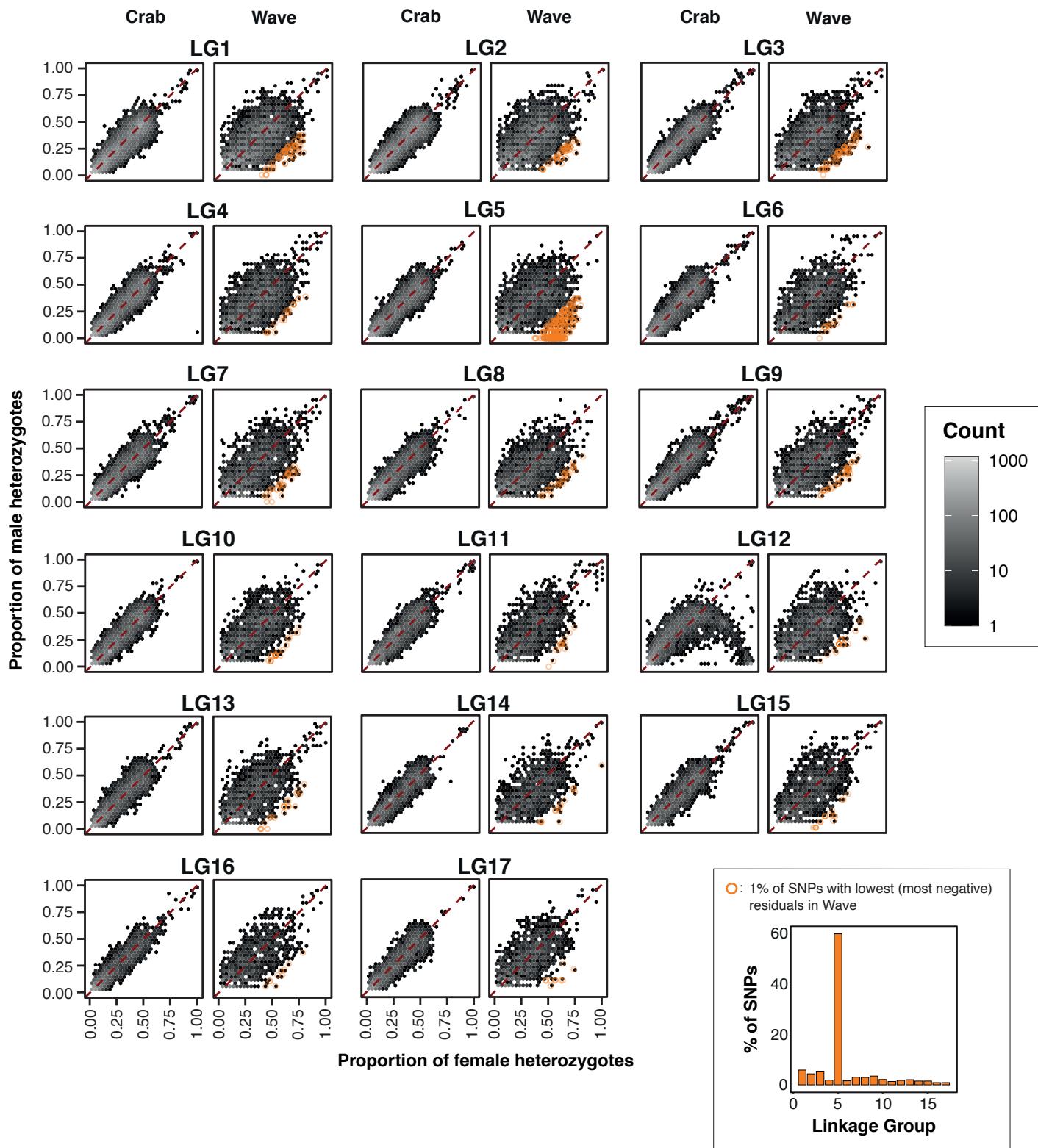

Supplement: Supplementary file 2 — Supporting Figure S1 [file EVL3-6-358-s004.pdf]

**A**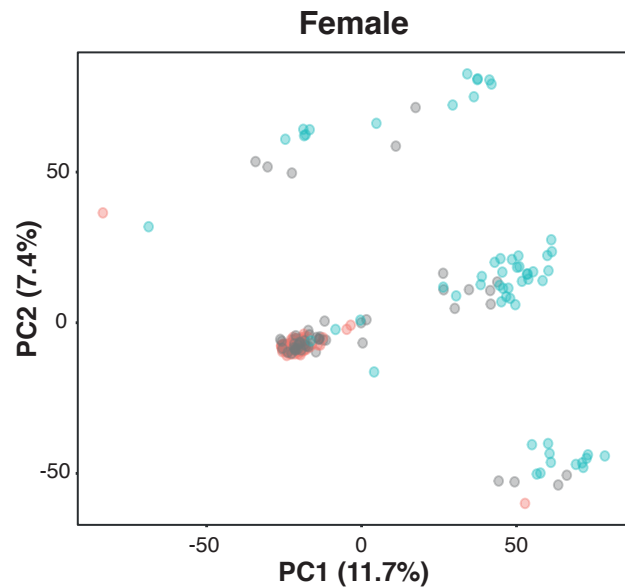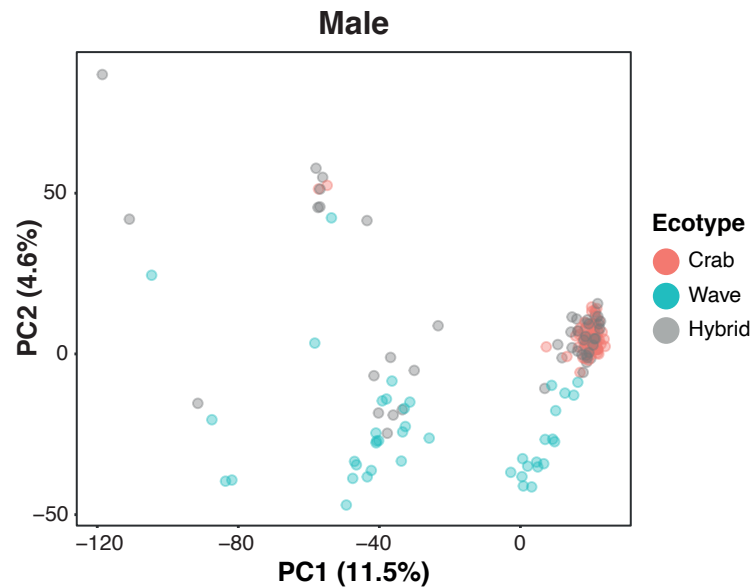**B**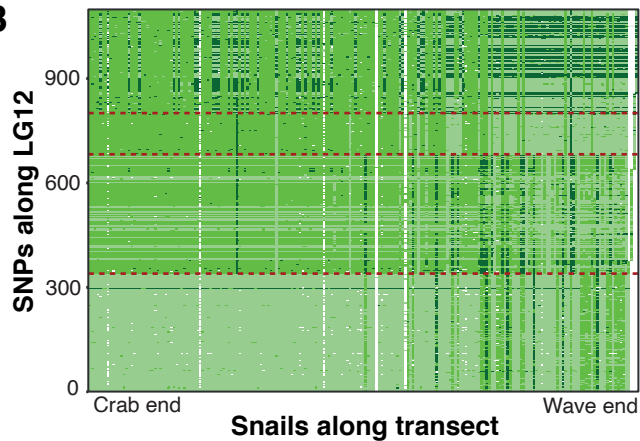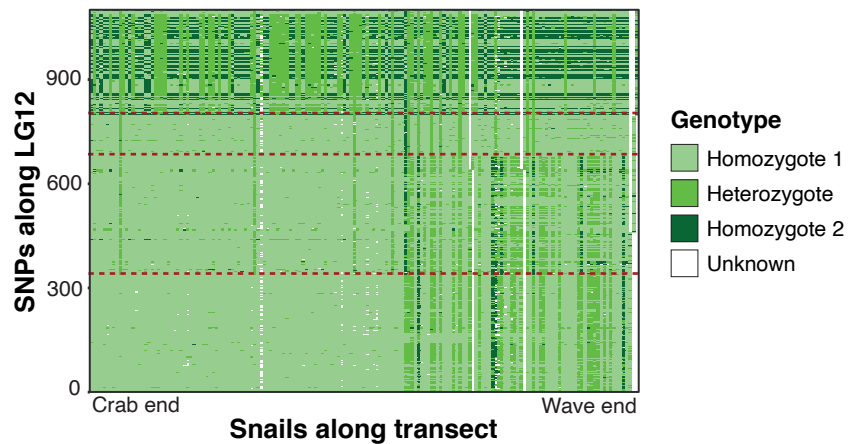

Supplement: Supplementary file 3 — Supporting Figure S2 [file EVL3-6-358-s002.pdf]

LGC12.1

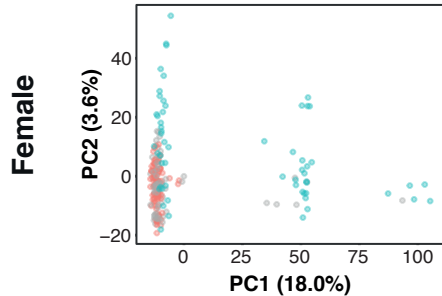

LGC12.2

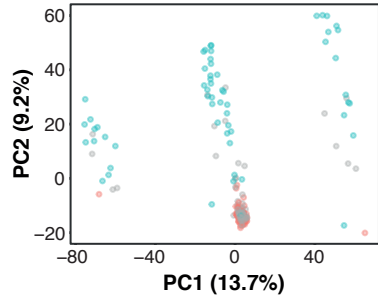

LGC12.3

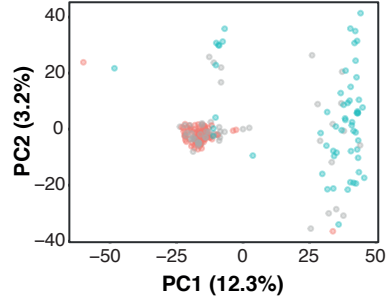

LGC12.4

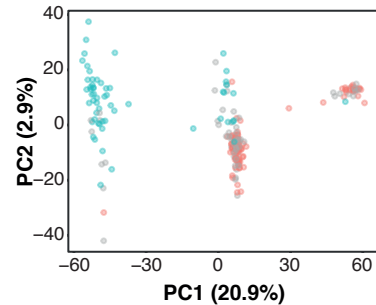

**Male**

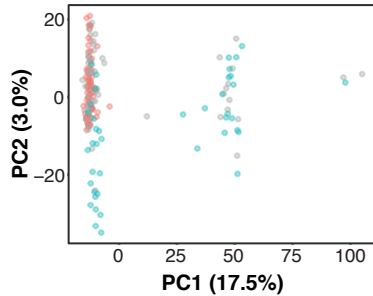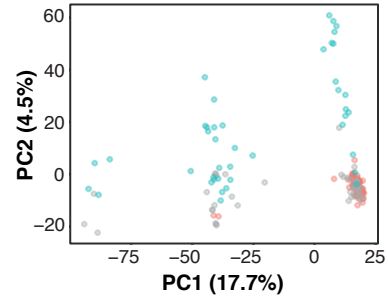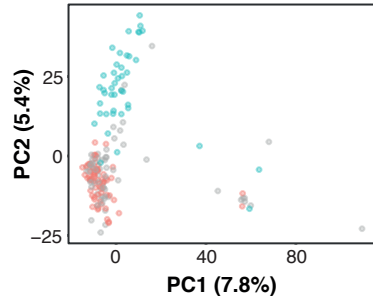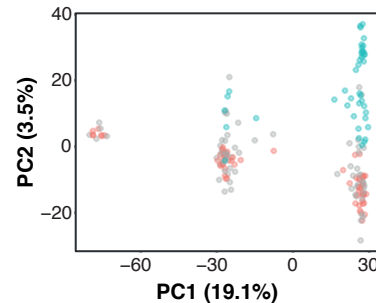

**Ecotype**

Crab Wave Hybrid

Supplement: Supplementary file 4 — Supporting Figure S3 [file EVL3-6-358-s001.pdf]

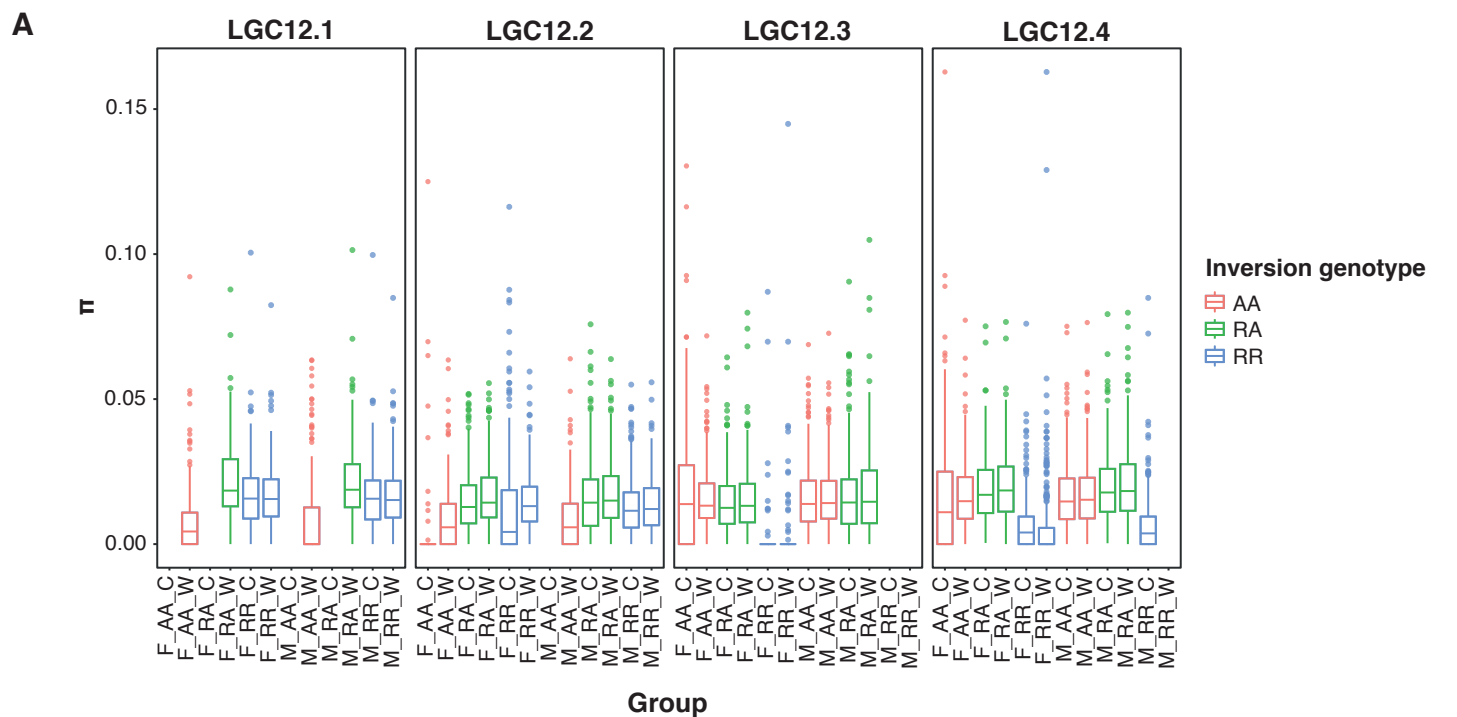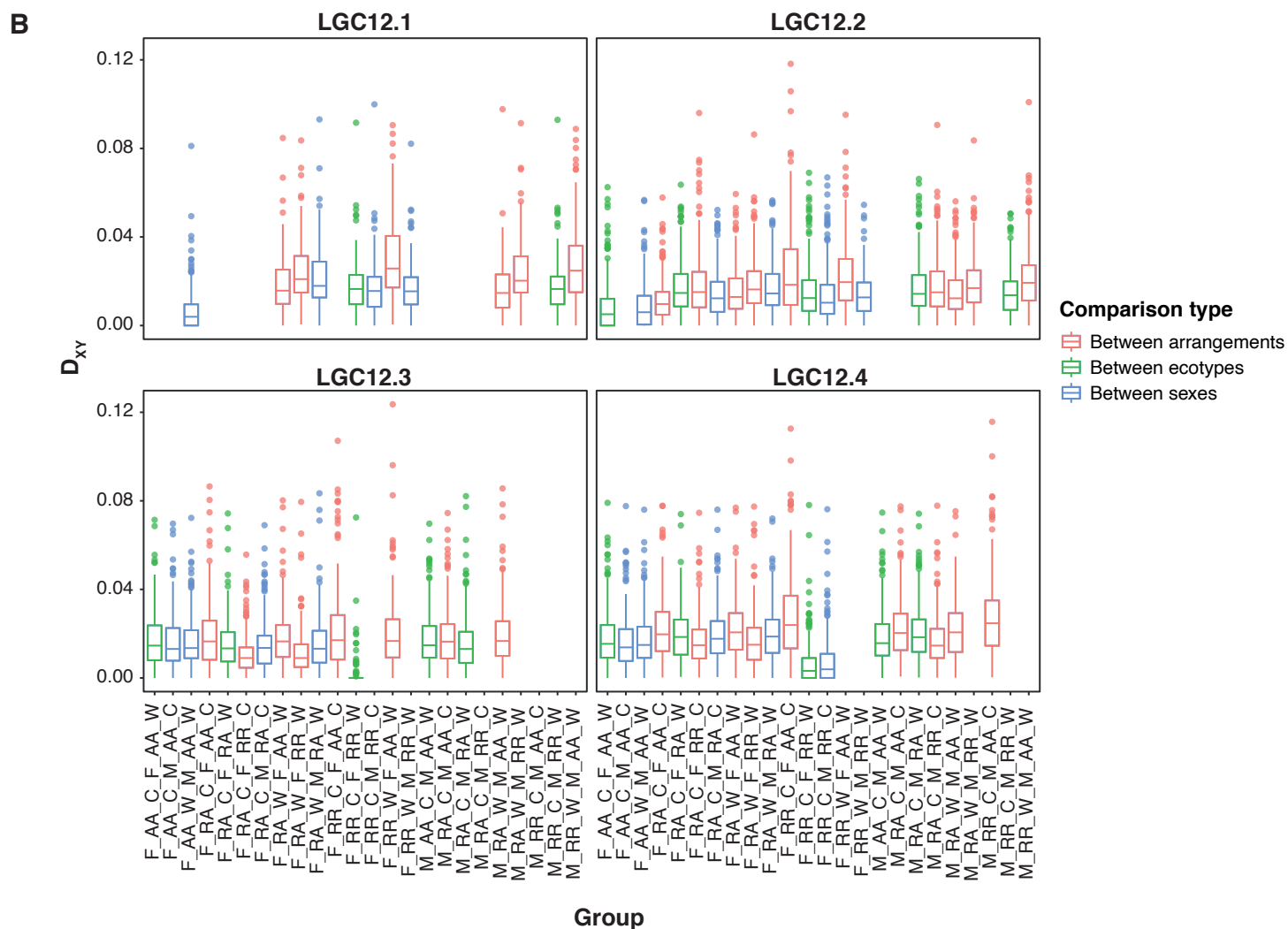

Supplement: Supplementary file 5 — Supporting Figure S4 [file EVL3-6-358-s005.pdf]
